# A genome-wide RNAi screen for genes important for proliferation of cultured *Drosophila* cells at low temperature identifies the Ball/VRK protein kinase

**DOI:** 10.1007/s00412-023-00787-6

**Published:** 2023-02-07

**Authors:** Anna Mendaluk, Emmanuel Caussinus, Michael Boutros, Christian F. Lehner

**Affiliations:** 1 Department of Molecular Life Science (DMLS), University of Zurich, Zurich, Switzerland; 2 Division of Signaling and Functional Genomics, German Cancer Research Center (DKFZ), Heidelberg, Germany; 3 Heidelberg University, BioQuant, Heidelberg, Germany

**Keywords:** Temperature acclimation, Ectotherms, Genome-wide RNAi screen, VRK protein kinase, Nuclear envelope, Chromosome condensation

## Abstract

A change in ambient temperature is predicted to disrupt cellular homeostasis by affecting all cellular processes in an albeit non-uniform manner. Diffusion is generally less temperature-sensitive than enzymes, for example, and each enzyme has a characteristic individual temperature profile. The actual effects of temperature variation on cells are still poorly understood at the molecular level. Towards an improved understanding, we have performed a genome-wide RNA interference screen with S2R + cells. This *Drosophila* cell line proliferates over a temperature range comparable to that tolerated by the parental ectothermic organism. Based on effects on cell counts and cell cycle profile after knockdown at 27 and 17 °C, respectively, genes were identified with an apparent greater physiological significance at one or the other temperature. While 27 °C is close to the temperature optimum, the substantially lower 17 °C was chosen to identify genes important at low temperatures, which have received less attention compared to the heat shock response. Among a substantial number of screen hits, we validated a set successfully in cell culture and selected *ballchen* for further evaluation in the organism. This gene encodes the conserved metazoan VRK protein kinase that is crucial for the release of chromosomes from the nuclear envelope during mitosis. Our analyses in early embryos and larval wing imaginal discs confirmed a higher requirement for *ballchen* function at temperatures below the optimum. Overall, our experiments validate the genome-wide screen as a basis for future characterizations of genes with increased physiological significance at the lower end of the readily tolerated temperature range.

**Supplementary Information:**

The online version contains supplementary material available at 10.1007/s00412-023-00787-6.

## Introduction

Many species thrive in thermally instable habitats. They cope with ambient temperature fluctuations using diverse strategies. A major distinction separates ecto- from endotherms. Cells in ectotherms operate essentially at the temperature of the environment. Although the term ectotherm is primarily used in animal biology, unicellular organism of all three domains of life (bacteria, archea, and eukaryota) as well as plants are also ectotherms in principle. In contrast, endotherms (primarily mammals and birds) invest metabolic energy for keeping their body temperature relatively high and constant. Importantly, however, this strategy does not abolish an exposure of cells to substantial temperature changes entirely, because temperature constancy is usually restricted to the body core. Thus, even in endotherms, cells in extremities or airways are not necessarily exempt from a need to operate at temperatures far from that of the body core.

The particularly challenging difficulties of operating over a range of temperatures result from the non-uniform effects of temperature fluctuations on the complex and diverse cellular structures and processes. Consequently, a change in ambient temperature is predicted to disrupt cellular homeostasis profoundly, and complex regulation is presumably required for a return to homeostasis. Beyond such basic considerations, our understanding of the actual effects of temperature change on cells remains limited. Research in temperature biology tends to focus on effects of extreme, evidently harmful temperatures, and a trend in favor of high over low temperatures was promoted by the early discovery of the heat shock response (Lindquist 1986; Ritossa 1962). In contrast, our study addresses effects of low temperatures that are still within the tolerated range.

In case of *Drosophila melanogaster*, which was used for our analyses, successful development to the adult stage occurs within the range from 11 to 32 °C. Often 25 °C is assumed to be the optimal temperature, but strain- and population-specific differences clearly exist. Compared to development, reproduction is more temperature-sensitive. Fertility of males is constrained to a more narrow temperature range from 12 to 30 °C (Chakir et al. 2002). Oviposition is observed between 11 and 34 °C, but it drops strongly below 14 and above 32 °C (Schnebel and Grossfield 1986). Propagation of *D. melanogaster* over many generation succeeds within the range of 14 to 29 °C (Petavy and Gibert 2001). Interestingly, cultured *D. melanogaster* cells appear to have a comparable temperature tolerance. S2R + cells (Yanagawa et al. 1998), the cell line used for our research, proliferate between 14 and 29 °C (Bai et al. 2021).

Cell lines derived from *D. melanogaster*, including S2R + cells, permit fast and efficient loss-of-function analyses based on RNA interference (RNAi) (Heigwer et al. 2018). *Drosophila* cells take up long double-stranded (ds) RNA molecules added into the growth medium very efficiently. Within the cell, dsRNA is processed into many different small-interfering RNAs, resulting in high knockdown efficiency of the targeted messenger (m) RNA. The ease of RNAi with *Drosophila* cells has fostered genome-scale functional screening for genes involved in a variety of biological processes, establishing the approach as a powerful and rewarding strategy in numerous research fields (Heigwer et al. 2018). One of the first genome-wide RNAi screens, which was performed in parallel with two cell lines (S2R + and Kc167), covered 91% of the annotated genes and identified those crucial for cell growth, survival, and proliferation based on an assay measuring ATP levels (Boutros et al. 2004). A similar screen with S2 cells and 70% coverage involved analysis by fluorescence-activated cell sorting (FACS) rather than imaging, revealing genes required for normal cell size and a normal cell cycle profile (Bjorklund et al. 2006).

To exploit genome-wide RNAi screening for the identification of genes that are of particular importance at low temperature, we have repeated the experimental schemes of Boutros et al. (2004) and Bjorklund et al. (2006), although with some decisive modifications. Most importantly, in comparison with the previous screens, we realized screening in a replicate format, where RNAi was applied in parallel in two replicates at 17 °C and in two additional replicates at 27 °C. High-throughput imaging after fluorescent labeling of microtubules and DNA was used for analysis of the RNAi effects, which included monitoring of cell cycle profiles obtained by quantification of the nuclear DNA contents. This screening approach identified genes of particular importance for manifestation of a normal cell cycle profile or a normal cell number at 17 °C, while less crucial or dispensable at 27 °C. A reduced cell number after knockdown of a particular gene can result in principle if the targeted gene promotes cell growth, progression through the cell cycle, or cell survival. In the following, genes characterized by reduced cell counts after depletion will be designated as cell proliferation genes for simplicity.

While genome-wide RNAi screening with S2R + cells is far less laborious than with flies, genes might possibly have distinct physiological significance in cultured cells and in the organism. For an initial evaluation of this issue, we selected one screen hit for further characterization in the organism. This gene, *ballchen* (*ball*), codes for a member of the metazoan VRK protein family, originally identified by a human expressed sequence tag coding for a product with amino acid sequence similarity to a vaccinia virus protein kinase (Nezu et al. 1997; Nichols et al. 2006; Zelko et al. 1998). The human homolog was thus named VRK1 (for vaccinia virus B1R kinase-related kinase 1) and two additional human family members as VRK2 and VRK3 (lacking kinase activity). *D. melanogaster* and *C. elegans* have a single family member most similar to mammalian VRK1 (Aihara et al. 2004; Gorjánácz et al. 2007). The *Drosophila* homolog was identified as a serine/threonine kinase present in extracts of early embryonic nuclei, which phosphorylated histone H2A but only when present in nucleosomes, explaining its naming as NHK-1 for nucleosomal histone kinase 1 (Aihara et al. 2004). The same gene was identified independently by molecular characterization of mutant alleles (Cullen et al. 2005; Herzig et al. 2014; Ivanovska et al. 2005), resulting in alternative names (*triplet* and *ball*). Complying with FlyBase nomenclature, *ball* will be used for the *D. melanogaster* VRK homolog. The phenotypic characterization of hypomorphic *ball* alleles revealed an involvement in nuclear organization in oocytes (Cullen et al. 2005; Ivanovska et al. 2005). In mid-stage oocytes, meiotic chromosomes normally lose their association with the nuclear envelope (NE) while compacting into a central dense chromatin clump. However, in hypomorphic *ball* mutants, the release of meiotic chromosomes from the NE is largely abolished. As a result, chromosome segregation during the meiotic divisions is severely defective, and embryos cannot develop. Null mutations of *ball* are associated with recessive lethality at the pupal stage (Cullen et al. 2005). Mitotically proliferating cells are strongly affected in *ball* null mutant larvae. Imaginal discs are missing or small; the brain is also small (Cullen et al. 2005). Clonal analyses in wing imaginal discs indicated that *ball*-mutant cells proliferate only to a limited extent after clone induction, followed by apoptotic elimination (Yakulov et al. 2014). The most prominent, best studied, and evolutionarily conserved function of mammalian VRK1 concerns also interactions between chromatin and NE and their regulation during progression through the cell cycle (Asencio et al. 2012; Cullen et al. 2005; Gorjánácz et al. 2007; Ivanovska et al. 2005; Molitor and Traktman 2014).

Analyses in mammalian cells (Nichols et al. 2006), *C. elegans* (Gorjánácz et al. 2007), and *D. melanogaster* (Lancaster et al. 2007) have demonstrated that a most crucial VRK substrate is the protein barrier of autointegration factor (BAF) (Lee and Craigie 1998; Sears and Roux 2020; Suzuki and Craigie 2002). BAF binds to dsDNA in a sequence-independent manner, as well as to LEM domains of proteins in the inner nuclear membrane (INM) and to Lamin A/C (Furukawa 1999; Segura-Totten and Wilson 2004; Shumaker et al. 2001). As a bridge between INM and DNA, BAF assists in the anchoring of chromatin to the nuclear periphery. Importantly, BAF’s interactions with INM and DNA are regulated by VRKs. Ball/VRK homologs phosphorylate BAF, resulting in a powerful inhibition of DNA binding (Gorjánácz et al. 2007; Lancaster et al. 2007; Marcelot et al. 2021; Nichols et al. 2006). Moreover, the interactions of BAF with LEM domains and lamins are also inhibited during M phase. Thus, at the onset of M phase, BAF can no longer anchor DNA at the NE periphery, resulting in the detachment of condensing chromosomes from the NE. Ball-driven inactivation of BAF also causes the detachment of meiotic chromosomes from the NE in the oocyte nucleus (Lancaster et al. 2007). Conversely, dephosphorylation of BAF during exit from M phase restores its DNA binding ability, supporting NE re-formation presumably by acting again as a bridge for the recruitment of membranes with INM proteins onto the de-condensing chromosomes (Haraguchi et al. 2001; Mehsen et al. 2018; Samwer et al. 2017). Beyond the control of NE-chromatin interaction, mammalian VRK1 appears to provide various additional chromatin regulatory functions (Campillo-Marcos et al. 2021). Similarly, *ball*^+^ was recently suggested to contribute to epigenetic gene regulation in *Drosophila* by supporting the activity of trxG proteins (Khan et al. 2021). Whether growth temperature affects the extent to which *ball*^+^ gene function is required has not yet been investigated.

According to our genome-wide screen, however, *ball*^+^ gene function is particularly important for S2R + cell proliferation at low temperature. The gene was among the screen hits associated with a most substantial difference in cell counts after knockdown at 17 and 27 °C, respectively. Our characterization in the organism confirmed that *ball* function is particularly crucial at low temperatures.

## Results

### Genome-wide RNAi screen for genes of temperature-dependent importance

**Fig. 1:**
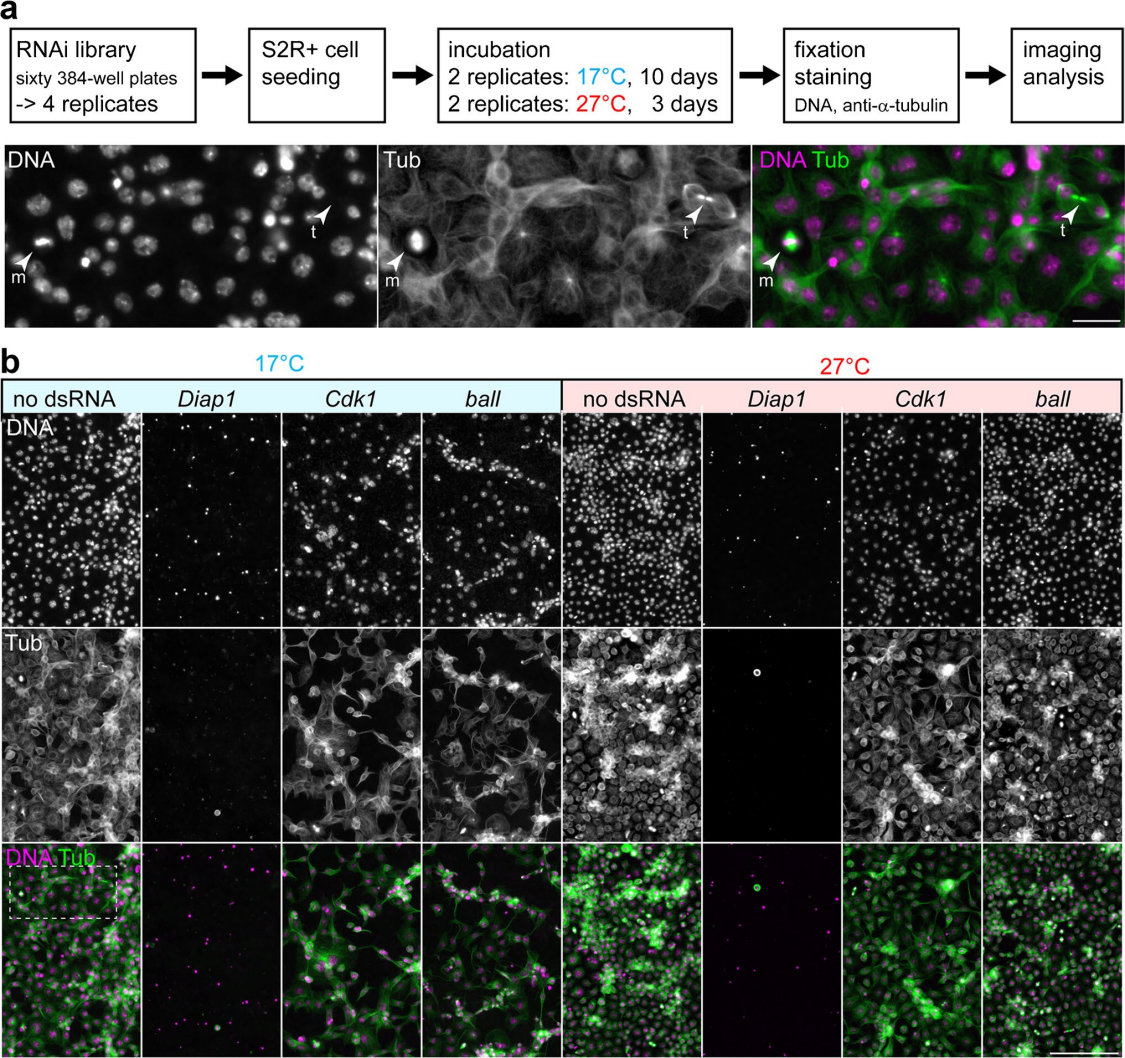
Genome-wide RNAi screen for genes of temperature-dependent importance. (**a**) Design of the RNAi screen. After exposure of S2R + cells to dsRNA at either 17 °C for 10 days or 27 °C for 3 days, cells were double labeled with a DNA stain (DNA) and green fluorescent anti α-tubulin (Tub) for image-based analysis. The images (bottom) display a region from a control well after incubation at 17 °C without dsRNA (see also dashed white rectangle in panel b) with mitotic figures indicated by arrowheads: metaphase (m) and telophase (t). (**b**) Representative examples for effects observed in the screen after treatment with the indicated dsRNAs for 10 days at 17 °C (left) or 3 days at 27 °C (right). Scale bars = 20 µm (a) and 100 µm (b)

To identify cell proliferation genes with a requirement that depends on growth temperature, we performed a genome-wide RNAi screen with *D. melanogaster* S2R + cells (Fig. 1a). These adherent cells are well suited for image-based screening. While usually cultured at around 25 °C, the presumed temperature optimum of the organism *D. melanogaster*, S2R + cells proliferate also at considerably lower temperatures, although with reduced cell spreading and increased aggregation (Bai et al. 2021). At 17 °C, S2R + cells are still fairly spread out. Moreover, at this temperature, an increase in cell numbers is readily detectable at day 10 after re-plating (Bai et al. 2021), and mitotic figures are also present at this time point (Fig. 1a). At 27 °C, a comparable increase in cell numbers required about 3 days according to our initial comparisons. Therefore, to compare the effect of knockdown of a particular gene at either 17 °C or 27 °C, RNAi treatment was done for 10 and 3 days, respectively, before cell analysis. During the screen, the cell number increase resulting at 27 °C was somewhat greater than that at 17 °C (Fig. 1b, compare no dsRNA at 17 °C and at 27 °C). RNAi appeared to be comparably effective in the two chosen conditions, as suggested by *Diap1* knockdown. *Diap1* codes for an essential anti-apoptotic factor, and its knockdown causes apoptosis (Boutros et al. 2004). The reduction in cell numbers after *Diap1* knockdown at either 17 °C for 10 days or 27 °C for 3 days was similar (Fig. 1b). The RNAi library HD2 was used for screening (Horn et al. 2010). This genome-wide library comprises sixty 384-well plates and targets 14,587 *D. melanogaster* transcripts. Four identical replicates with dsRNA aliquots from the library were generated (Fig. 1b). Two replicates were used for S2R + cell treatment at 17 °C and two at 27 °C. For image-based analysis after dsRNA treatment, we performed double labeling with a DNA stain and a green fluorescent antibody against α-tubulin (Fig. 1b).

The final images obtained in the screen are illustrated with a few characteristic examples (Fig. 1b). Wells without any dsRNA (negative controls) contained a relatively high number of cells at both temperatures (Fig. 1b). As already stated above, knockdown of *Diap1* resulted in a strong reduction of cell counts at both temperatures (Fig. 1b). Similarly, a reduction in cell numbers at both temperatures although to a lesser extent resulted after knockdown of *Cdk1* (Fig. 1b), which is essential for progression through the cell cycle (Stern et al. 1993). In contrast, knockdown of some genes, including *ballchen* (*ball*), had an effect that was clearly dependent on temperature. In case of dsRNA targeting *ballchen* (*ball*), cell number was more drastically reduced at 17 compared to 27 °C (Fig. 1b).

**Fig. 2:**
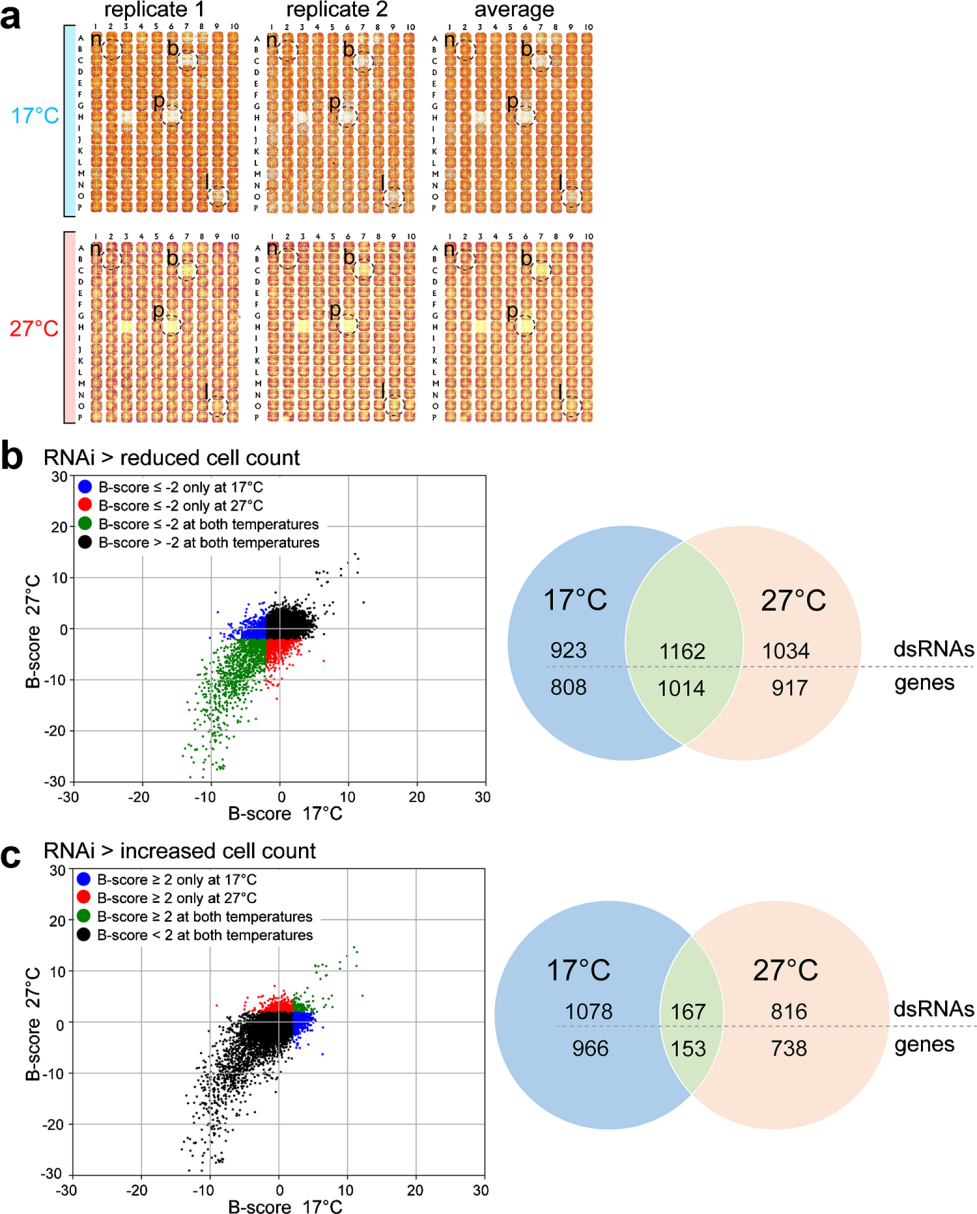
RNAi screen hits with temperature-dependent effects on cell numbers. (**a**) Overall DNA content in wells revealed by low-magnification images of the DNA signals. The equivalent regions of 384-well plates of all four replicates, as well as an average image of the two same-temperature replicates are displayed. Dashed circles indicate a negative control well without dsRNA (n), a positive control well with *Diap1* dsRNA (p), a well with reduced overall DNA content at both temperatures (b), and a well with low DNA predominantly at the low temperature (l). (**b**, **c**) Scatter plots (left) display the cell count B-scores (mean B-score of the two same-temperature replicates) after knockdown at either 17 °C or 27 °C for all the tested dsRNA amplicons. Venn diagrams (right) indicate the numbers of dsRNA amplicons classified based on the temperature dependency of their effects on cell counts. The number of genes identified by these dsRNA amplicons is given below the dsRNA amplicon number. (**b**) Values of dsRNA amplicons resulting in unusually low cell numbers (average B-score ≤  − 2) are colored in case of amplicons associated with reduced cell counts at only 17 °C (blue), at only 27 °C (red), and at both temperatures (green). (**c**) Values of dsRNA amplicons resulting in unusually high cell numbers (average B-score ≥  + 2) are colored in case of amplicons associated with increased counts at only 17 °C (blue), only 27 °C (red), and at both temperatures (green)

For an initial evaluation of the technical quality of the screen, we used low magnification images of the DNA signals, which revealed an obvious reproducibility between replicates, for example, for the negative and positive control wells (Fig. 2a). These low magnification images were also used for an initial identification of genes with a requirement that appeared to be temperature-dependent. For this initial screen hit identification, we averaged the images of the two same-temperature replicates. Visual inspection of the resulting average images confirmed the presence of some wells with a reduction of the overall DNA content predominantly at only one of the two analyzed temperatures (Fig. 2a).

For a more quantitative hit identification, cell counts were determined for each well by automated analysis of high magnification images. B-scores (Brideau et al. 2003) of the obtained cell counts were calculated (Online Resource 1, S1 Table). Analysis of the B-score distribution indicated that the screen data was not distorted by severe technical artefacts affecting certain plates or replicates (Online Resource 2, S1 Figure). The reproducibility of B-scores obtained for the two 27 °C replicates was higher overall than in case of the two 17 °C replicates (Online Resource 2, S1 Figure). Although technical reasons for the lower reproducibility at the low temperature are not excluded, it might also reflect increasing phenotypic decanalization at temperatures further away from the optimum, as proposed in case of transcriptome changes in adult *D. melanogaster* (Chen et al. 2015). Compared to the overall correlation between the two 27 °C replicates (R^2^ = 0.63), the correlations between 27 and 17 °C replicates were lower (R^2^ = 0.44, 0.37, 0.47, and 0.39 for 27_repl1 vs 17_repl1, 27_repl1 vs 17_repl2, 27_repl2 vs 17_repl1, and 27_repl2 vs 17_repl2, respectively). However, the correlations between 27 and 17 °C replicates were not consistently lower in comparison to the overall correlation between the two 17 °C replicates (R^2^ = 0.39).

Because only two replicates per temperature were analyzed in the genome-wide RNAi screen, further validation of screen hits appeared to be crucial. To identify screen hits that might deserve further validation, we focused on dsRNA amplicons resulting in cell counts that were either unusually low or unusually high (B-score of cell count ≤ -2 or ≥  + 2). About 25% of all analyzed dsRNA amplicons resulted in such unusual cell counts, in case of all four replicates. Unusually low cell counts were observed about two-fold more often than unusually high cell counts, consistent with the notion that genes required for growth, proliferation, or survival of S2R + cells are more numerous than genes with negative effects on these processes, as clearly confirmed for yeast (Yoshikawa et al. 2011).

In a second step, we selected dsRNA amplicons that affected cell counts differentially at the two analyzed temperatures (Fig. 2) and generated several initial hit lists (Online Resource 3, S2 Table).

A first hit list, l17weak, contained genes associated with unusually low cell numbers after knockdown at 17 °C (average B-score ≤ -2), while cell counts were at most slightly reduced at 27 °C. Eight hundred nine genes were on this list, identified by 923 distinct dsRNA amplicons resulting in weak cell proliferation preferentially at 17 °C. A similar list (l17weak_a: 150 genes identified by 162 dsRNA amplicons) was generated from amplicons resulting in a reduction of cell counts at both 17 and 27 °C but far more extensively at the lower temperature. These two lists contained candidate genes more important for cell cycle progression, growth, and survival at low temperature. A third list, l27weak, contained genes of opposite character, i.e., greater importance at the higher, near-optimal temperature. This list contained 917 genes identified by the 1034 dsRNA amplicons that were associated with unusually low cell numbers after knockdown at 27 °C (average B-score ≤ -2) but not after knockdown at 17 °C.

Two additional lists were generated with hits characterized by the opposite, i.e., unusually high rather than low cell numbers at only one of the two tested temperatures. In case of l27strong, cell counts were unusually high at 27 °C; it comprised 738 target genes identified by 816 distinct dsRNA amplicons. The list l17strong contained 966 genes identified by 1078 unique dsRNA amplicons resulting in unusually high number of cells at 17 °C.

Candidate genes important at both temperatures were also identified for comparison with the candidate genes of differential importance at the two analyzed temperatures. Cell counts were unusually low at both temperatures after knockdown of 1014 genes identified by 1162 distinct dsRNA amplicons (average B-score ≤  − 2 at both 17 and 27 °C, see l_both_weak). These 1014 genes of non-temperature-dependent importance were slightly more than the genes primarily important at 17 °C (809) or primarily important at 27 °C (917) (Fig. 2a). In contrast, candidate genes associated with unusually high cell numbers after knockdown at both temperatures (average B-score ≥  + 2 at both 17 and 27 °C, see l_both_strong) were far less numerous than those of differential importance (Fig. 2b).

### Validation of selected candidate genes of increased importance at low temperature

A selection of genes of increased importance at low temperature according to the genome-wide screen was validated with additional RNAi experiments. Candidate genes were chosen based on various criteria. Preference was given to strong hits, i.e., those with an extensive difference between cell counts at 17 and 27 °C, as well as low variability between the two same-temperature replicates. However, strong hits were only considered in case of clear evidence for expression in S2R + cells according to RNA-Seq data (Brown et al. 2014). Finally, genes without functional annotations were given low priority.

**Fig. 3:**
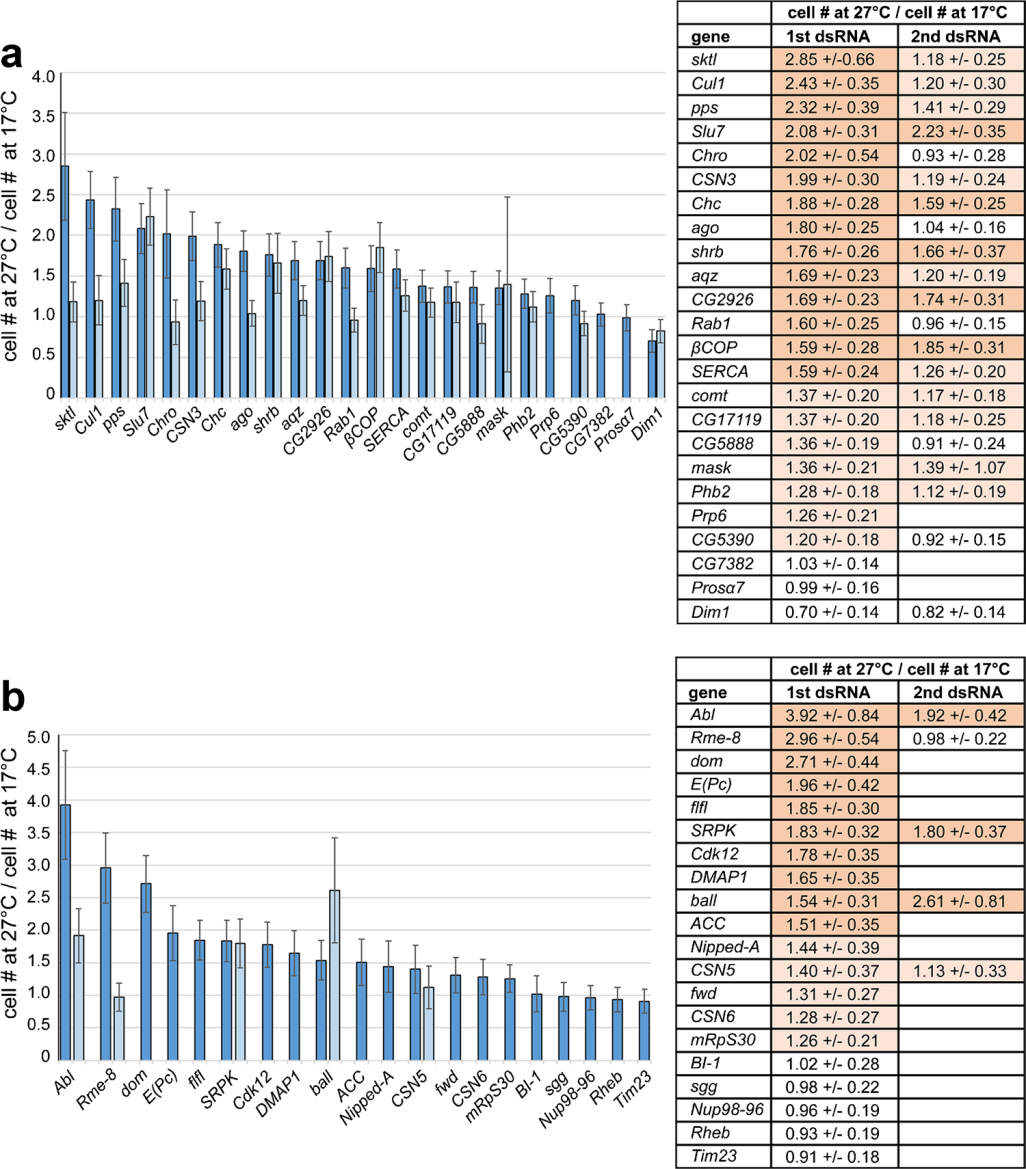
Validation of screen hits associated with low cell counts after knockdown especially at 17 °C. Candidate genes suggested to be of increased importance at low temperature by the genome-wide RNAi screen were selected based on either overall DNA signal intensities in low-magnification images (**a**) or cell counts according to high-magnification images (**b**). For validation experiments, RNAi was induced either with the same dsRNA amplicon as in the screen (dark bars, 1st dsRNA) or with a second distinct dsRNA amplicon (light bars, 2nd dsRNA). After addition of dsRNA, S2R + cells were cultured at either 17 °C for 10 days or 27 °C for 3 days before determination of cell counts. Ratio of cell counts at 27 °C and 17 °C in the tables are marked with light shading when greater than 1.1 and dark shading when greater than 1.5. See Materials and Methods for explanations concerning normalization of cell counts, error bars, and number of replicates

An initial set of 24 candidate genes, selected early on based on the visual comparison of the overall DNA content in the low magnification images (Fig. 2a), was characterized by low cell numbers specifically at 17 °C. The subsequent quantitative cell count analyses with high-resolution images also identified all these genes as of higher importance at low temperature (i.e., they were present in the l17weak or the l17weak_a hit lists) except for five (*Prosα7*, *Prp6*, *Phb2*, *CG5390*, *Cul1*). For experimental validation of the selected 24 candidate genes, an additional RNAi experiment was completed, and knockdown effects at 17 and 27 °C were again compared. For this validation experiment, the same dsRNA amplicon as in the genome-wide screen was used, but the dsRNA was independently prepared. Moreover, some technical aspects of the validation experiments were different (scale of cell culture, image acquisition, and analysis, see “Materials and Methods”). The validation experiment confirmed the results of the screen for 21 of the 24 selected genes at least qualitatively (Fig. 3a). After knockdown of these 21 genes, a lower number of cells were detected at 17 compared to 27 °C. For 14 of these 21 genes, the 27 °C/17 °C cell count ratio was higher than 1.5 (Fig. 3a). The 21 confirmed genes were subject to a second round of validation using distinct dsRNA amplicons for RNAi. In this second experiment, lower cell numbers at 17 compared to 27 °C resulted in case of 15 out of the 21 genes, but a 27 °C/17 °C cell count ratio > 1.5 was observed for only 5 genes (Fig. 3a).

A second set of candidate genes was selected based on the cell count analysis with high-resolution images (Fig. 2b). In total, 20 additional candidates were selected, 11 from the list l17weak and 9 from the list l17weak_a. This second set of candidate genes was also analyzed in an RNAi experiment with an independently produced dsRNA preparation of the same amplicon used before in the screen. For a few candidates, also a distinct additional dsRNA amplicon was used. For 15 of the 20 genes, cell counts were lower at 17 compared to 27° after knockdown with the screen dsRNA amplicon (Fig. 3b). Ten of the 15 genes were characterized by a 27 °C/17 °C cell count ratio > 1.5. In case of the five genes that were further characterized with a second independent dsRNA amplicon, four were observed to have fewer cells at the low temperature, and three had a 27 °C/17 °C cell count ratio > 1.5 (Fig. 3b).

In summary, the validation experiments confirmed the screen results qualitatively for 80% of the selected candidate genes (*n* = 44) when the same dsRNA amplicon was used for screening and validation. However, the temperature-dependency of cell numbers after knockdown was modest for several candidate genes in the validation experiments. Considering only those with a validated 27 °C/17 °C cell count ratio > 1.5 reduced the confirmation rate to 50%. As the genes were not randomly chosen for validation, these confirmation rates cannot be extrapolated to all genes included in the hits lists. However, in case of strong hits, the screen results appear to be reproducible in 50% of the cases. Additional validation with a second distinct dsRNA amplicon resulted in a further reduction of the confirmation rate. About 42% of the genes (8 out of 19), which had a 27 °C/17 °C cell count ratio > 1.5 in the re-test with the screen dsRNA amplicon, had again a 27 °C/17 °C cell count ratio > 1.5 when analyzed with a second distinct dsRNA amplicon. A failure of confirmation with a second distinct dsRNA amplicon might reflect either off-targets effects of the first dsRNA amplicon or insufficient knockdown efficiency of the second dsRNA amplicon. Overall, we conclude that our genome-wide RNAi screen provides a highly useful basis for selection of candidate genes of temperature-dependent importance for further detailed functional characterization.

### Cell cycle profile analysis for genome-wide identification of genes of temperature-dependent importance

**Fig. 4:**
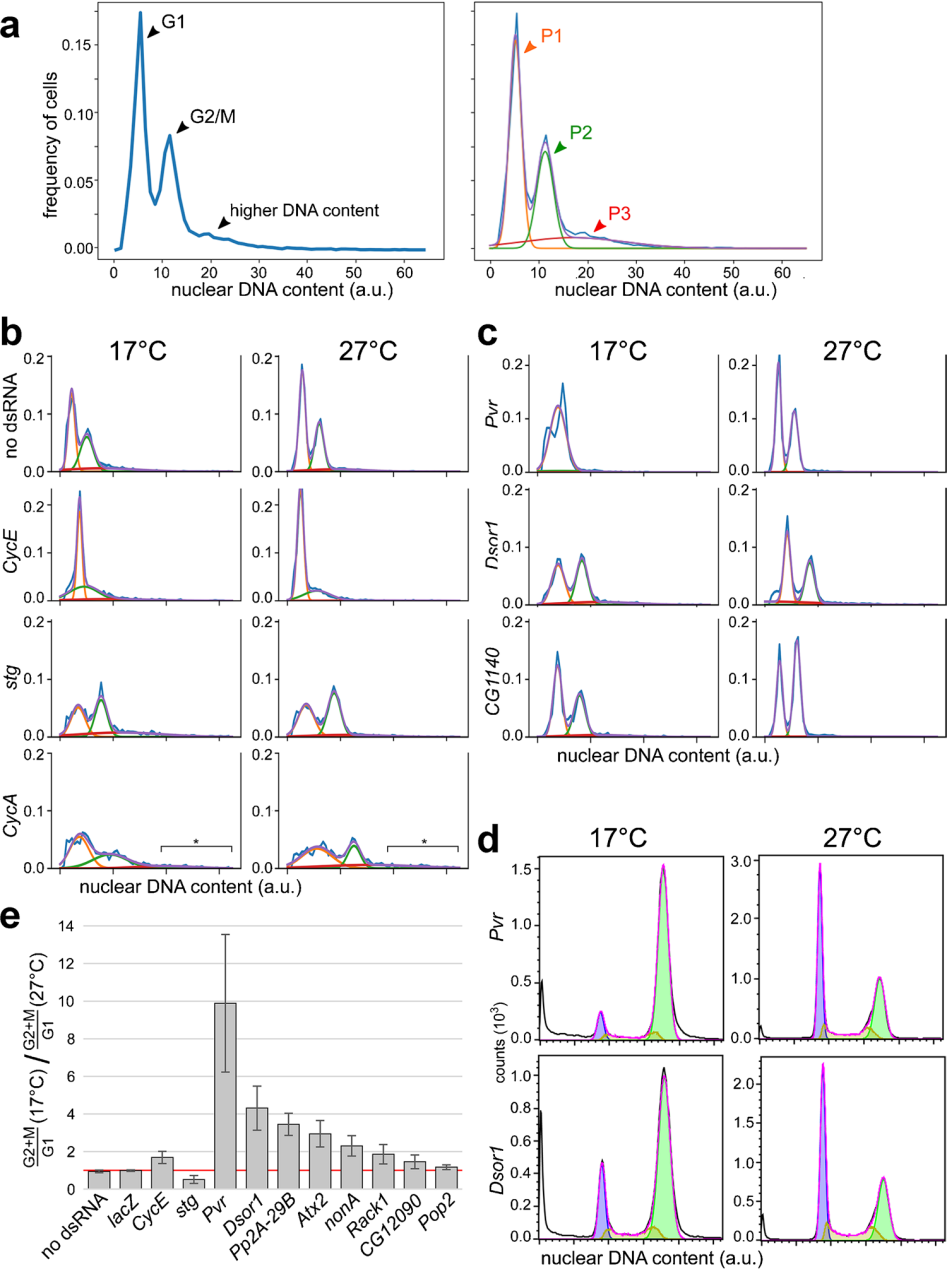
Identification of genes of temperature-dependent importance based on cell cycle profiles. (**a**) Integrated DNA signal intensity was determined for each nucleus in a given well, allowing the generation of histograms, i.e., cell cycle profiles (left). The characteristic cell cycle profile from a negative control well is displayed. A model with three distinct Gaussian cell populations was fitted to the observed cell cycle profile (right). The three populations correspond largely to the G1 cells (P1, orange), to the G2/M cells (P2, green), and to the abnormal cells with higher ploidy (P3, red). The cell cycle profile resulting after model fitting is shown in purple and the observed cell cycle profile in blue. (**b**, **c**) Comparison of cell cycle profiles observed after knockdown at 17 and 27 °C, respectively, with the indicated dsRNAs. (**b**) Minimal temperature dependence was observed in negative control wells and after knockdown of *bona fide* cell cycle regulators. The enrichment of cells with increased ploidy after knockdown of *CycA* at 17 and 25 °C is indicated (asterisk and bracket). (**c**) Examples with abnormal cell cycle profiles generated after knockdown at only one of the two analyzed temperatures. (**d**, **e**) Validation of the indicated candidate genes important for a normal cell cycle profile at 17 °C but not 27 °C. (**d**) After knockdown at either 17 or 27 °C, the cell cycle profile was determined by flow cytometry, as shown for *Pvr* and *Dsor1*. (**e**) The temperature dependence of the cell cycle profile was quantified by calculating the ratio indicated along the x-axis. Absence of temperature dependence result in a value of 1 (red line)

The high-content image data acquired in our RNAi screen offers the possibility to use readouts other than cell counts for the identification of genes with a temperature-dependent requirement. Cell counts are not necessarily a most sensitive readout given the design of our screen assay. The number of S2R + cells increased around fivefold at most during the 3-day incubation period at 27 °C used in the RNAi screen. This increase in cell number cannot be completely prevented, even if a given dsRNA effectively targets a gene essential for cell cycle progression, because depletion is not instantaneous. If effective depletion takes about 3 days, a reasonable estimate for many genes, there cannot yet be an effect on cell counts in our screening assay. The same reasoning also applies for depletion at 17 °C. However, compared to cell counts, significant alterations in the cell cycle profile might arise more rapidly after depletion for some genes. Cells might already be largely arrested, in the G1 phase, for example, while still comparable to controls in number at the time of fixation. Therefore, to identify additional genes of increased importance at low temperature, which might have escaped detection based on cell counts, we performed further screen data analyses on cell cycle profiles. Image-based quantitative analysis of the nuclear DNA signal intensities allowed the generation of histograms of DNA signal intensity per nucleus, i.e., the cell cycle profile of the cells in a given microtiter plate well (Fig. 4a). For extraction of quantitative parameters of the cell cycle profile, a cell population model was fitted to the DNA signal intensity histograms. This model assumes that the cell population within a well is composed of three distinct Gaussian sub-populations. In unperturbed negative control cells (Fig. 4a), the first sub-population (P1) corresponds approximately to the cells in the G1 phase of the cell cycle, the second sub-population (P2) to the G2/M cells, and third sub-population (P3) to abnormal hyperploid cells, which are rare in unperturbed cells. In the large majority of the wells analyzed in the genome-wide RNAi screen, the DNA signal intensity histogram and the fitted cell population model was essentially identical to that observed in the negative control wells (Fig. 4 a and b and Online Resource 1, S1 Table). In contrast, inspection of the cell cycle profile of wells treated with dsRNAs depleting well-characterized cell cycle regulators, for example, Cyclin E (CycE), String (Stg)/Cdc25 phosphatase, and Cyclin A (CycA) (Fig. 4b), clearly revealed the expected changes. A massive enrichment of G1 cells was observed after depletion of CycE (Fig. 4b), and depletion of Stg/Cdc25 phosphatase resulted in an enrichment of G2/M cells (Fig. 4b). In case of CycA, which has been demonstrated to result in endoreduplication in the closely related S2 cells after more extended depletion (Rotelli et al. 2019), the onset of endoreplication was detectable (Fig. 4b). Thus, abnormalities in the cell cycle profile can be detected readily by analysis of our high magnification images.

The knockdown effects in case of *bona fide* cell cycle regulators (like CycE, Stg/Cdc25, and CycA) were observed at both 17 and 27 °C (Fig. 4b). Interestingly, however, there were also genes, where knockdown resulted in an altered cell cycle profile predominantly at only one of the two temperatures, at either at 17 °C in case of *Pvr* and *Dsor1* or at 27 °C in case of *CG1140* (Fig. 4c).

For identification of genes, for which knockdown resulted in an altered cell cycle profile predominantly at either the low or the high temperature, we generated hit lists using the data extracted after fitting of the sub-population model (see “Materials and Methods”). The primary purpose of these lists (Online Resource 4, S3 Table) was again assistance in the selection of candidate genes to be validated with additional experiments. Eight candidate genes were selected for validation, focusing on those primarily important at the low temperature. Only strong hits with clear evidence for expression in S2R + cells were chosen. For validation, we completed an RNAi experiment analogous to that used for genome-wide screening. The same dsRNA amplicon was used for RNAi as in the screen but with independently generated dsRNA. After cell culture at a larger scale, flow cytometry was used for analyses rather than imaging as in the screen. Beyond negative control experiments (no dsRNA and *lacZ* dsRNA), several positive controls (dsRNA of *CycE*, *stg*, and *Cdk1*) were included. The cell cycle profiles obtained with the negative and positive control dsRNAs were analogous to those observed in the screen, and importantly they were very similar at 17 and 27 °C (Online Resource 5, S2 Figure). To quantify the effect of temperature on the cell cycle profiles, we determined first the ratio of the size of the G1 and of the G2/M sub-population. These sub-population ratios were determined for the 17 °C and for the 27 °C condition. Thereafter, these rations were compared by quotient formation (Fig. 4e). The resulting 17 °C/27 °C measure was essentially 1 when no dsRNA or *lacZ* dsRNA was added for RNAi (Fig. 4e), indicating a near identity of the cell cycle profiles at the two analyzed temperatures. In case of *CycE* dsRNA, the ratio was 1.69 (Fig. 4e), because the G1 arrest was slightly less pronounced at 17 °C. In case of *stg* dsRNA, the ratio was 0.51, because the G2 arrest was slightly less pronounced at 17 °C (Fig. 4e). In comparison to the controls, most of the candidate genes were characterized by knockdown effects on the cell cycle profile that were far more temperature-dependent (Fig. 4e). As already in the RNAi screen, knockdown of these candidate genes resulted in an enrichment of G2/M cells that was more pronounced at 17 compared to 27 °C. The most pronounced temperature effects were observed for *Pvr* and *Dsor1* (Fig. 4 d and e), while it was minimal in case of *Pop2* (Fig. 4e).

The abnormal cell cycle profile resulting after knockdown of *Pvr* and *Dsor1* at 17 °C might reflect a cell cycle arrest during either G2- or M phase. Alternatively, it might arise from a cytokinesis failure starting relatively late during the RNAi treatment period. Indeed, the cell cycle profiles after knockdown of *Pvr* and *Dsor1* at 17 °C were not just similar to the cell cycle profile resulting from knockdown of the M phase inducer *stg/cdc25 phosphatase* (Edgar and O'Farrell 1990) but also to that obtained after knockdown of *pbl* (Online Resource 6, S3 Figure), which is essential for cytokinesis (Lehner 1992). For further insight into the cell-cycle arrest resulting from knockdown of *Pvr* and *Dsor1* at 17 °C, we inspected the images obtained in the screen. Images acquired after *pbl* knockdown revealed many binucleate cells, as expected (Online Resource 6, S3 Figure). In contrast, knockdown of *Dsor1* and *Pvr* at 17 °C did not result in an increased presence of multinucleated cells. Moreover, the cells had an interphase appearance, excluding an M phase arrest. Finally, cell cycle profiles and images also differed from those resulting after *CycA* knockdown, arguing against endoreduplication. In conclusion, knockdown of *Pvr* or *Dsor1* at 17 °C induces a cell cycle arrest during the G2 phase.

Overall, the analyses based on cell cycle profiles demonstrated that this permits a sensitive identification of genes that are important for normal cell cycle progression primarily at low temperature.

### Increased requirement for Ball/VRK protein kinase at low temperature during early embryonic mitoses

Genome-wide RNAi screening with S2R + cells is less laborious than with flies, but the physiological significance of a given hit is not necessarily identical in cultured cells and in the organism. For further evaluation in the organism, we focused on the gene *ballchen* (*ball*), which was particularly important for S2R + cell proliferation at low temperature. *ball*, which codes for the *Drosophila* VRK protein kinase homolog, was among the screen hits associated with a most substantial difference in cell counts after knockdown at 17 and 27 °C, respectively. Four distinct *ball* dsRNA amplicons resulted in a far greater reduction in cell numbers at 17 compared to 27 °C (mean B score of the four amplicons at 17 °C =  − 4.48 and at 27 °C =  − 0.25). Moreover, the functions previously attributed to *ball* were in line with low-temperature effects exposed by time-lapse imaging with early *D. melanogaster* embryos. On the one hand, this earlier time-lapse imaging (Radermacher 2012) had suggested that the detachment of chromosomes from the nuclear envelope (NE) during condensation at the start of mitosis is sensitive to low temperature. On the other hand, Ball/VRK is known to regulate the release of chromosomes from the NE at optimal temperature (Asencio et al. 2012; Cullen et al. 2005; Gorjánácz et al. 2007; Ivanovska et al. 2005; Molitor and Traktman 2014).

**Fig. 5:**
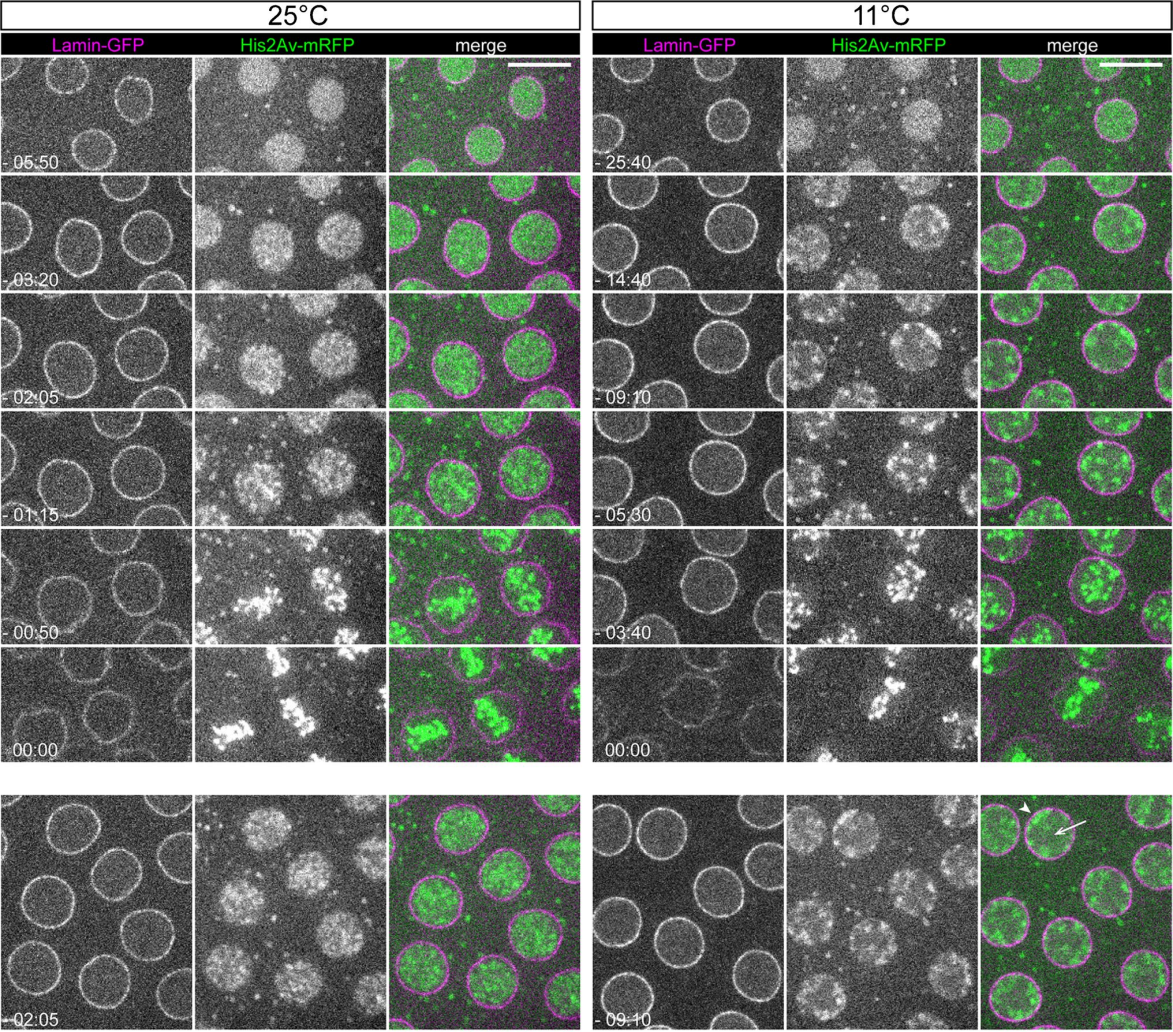
Low temperature alters the pattern of chromosome condensation in early embryos. Syncytial blastoderm embryos expressing Lamin-GFP and His2Av-mRFP were analyzed by time-lapse imaging at 25 °C and 11 °C, respectively. Single optical sections with nuclei progressing into M12 are displayed. Time (min:sec) is indicated with *t* = 0 corresponding to the first frame that displayed the final metaphase plate. The bottom row presents larger fields with additional nuclei at the time point when the temperature induced difference in the spatial pattern of the initial chromosome condensation is most clearly apparent. At 25 °C, condensing chromosome detaches immediately from the nuclear periphery and contracts towards the interior. However, at 11 °C, chromosome detachment from the NE occurs with a delay, and thus the interior (arrow) is comparatively devoid of condensing chromosome, and the periphery is characterized by foci of associated condensing chromosomes (arrowhead). Scale bar = 10 μm

To corroborate the temperature sensitivity of chromosome detachment (Radermacher 2012), we performed additional time-lapse imaging followed by quantitative analyses (Fig. 5). Embryos expressing Lamin-GFP to visualize the nuclear lamina and His2Av-mRFP to visualize chromosomes were analyzed at the presumed optimal temperature (25 °C) and at a low temperature (11 °C). The analysis was focused on progression through nuclear cycle 12, which occurs during the syncytial blastoderm stage. This stage is characterized by the presence of a monolayer of syncytial nuclei just underneath the plasma membrane, a position most optimal for microscopic analysis. Lamin-GFP is an excellent marker for the nuclear periphery not only during interphase but also during the first half of mitosis, because the nuclear lamina depolymerize slowly and incompletely in *D. melanogaster*. During interphase, His2Av-mRFP signals were indistinguishable at 25 °C and 11 °C. At both temperatures, these signals were distributed rather uniformly throughout the nucleus (Fig. 5). During chromosome condensation at the onset of mitosis 12 (M12), His2Av-mRFP signals increasingly lost their homogenous nuclear distribution. At 25 °C, the condensing chromosomes receded rapidly from the nuclear periphery and contracted towards the interior (Fig. 5). In contrast, at 11 °C, the condensing chromosome remained closely attached to the nuclear periphery at several focal points (Fig. 5). Chromosome detachment from the nuclear periphery occurred eventually also at 11 °C, with a clear delay compared to 25 °C. A metaphase plate was therefore formed at both temperatures (Fig. 5), followed by a normal exit from M phase.

The delayed chromosome detachment at the onset of mitosis in syncytial blastoderm embryos observed at 11 °C supported the notion that the requirement for *ball* function, which promotes the swift release of condensing chromosomes from the nuclear periphery (Asencio et al. 2012; Cullen et al. 2005; Gorjánácz et al. 2007; Ivanovska et al. 2005; Molitor and Traktman 2014), may be particularly high at low temperature. Accordingly, a partial reduction of *ball* function is predicted to delay the release of condensing chromosomes from the nuclear periphery most strongly at low temperatures. To assess this prediction, we analyzed embryos collected from females with either one or two functional *ball*^+^ gene copies. Progression through early embryogenesis, including cycle 12 of the syncytial blastoderm stage, is supported by maternal mRNAs and proteins, as the major activation of zygotic gene transcription proceeds later during cellularization. Therefore, it is the maternal *ball* genotype that determines how much *ball* function is present during early embryogenesis. Embryos were collected from mothers heterozygous for the null allele *ball*^2^ (Herzig et al. 2014), and these embryos will be designated as *ball*^+^_1 in the following. For comparison, *ball*^+^_2 embryos were collected from *ball*^2^ heterozygous mothers that also carried a copy of *g-ball*, a transgene with a genomic fragment that promotes full rescue of *ball* null mutant flies (Herzig et al. 2014). The mothers of both *ball*^+^_1 and *ball*^+^_2 embryos provided them also Lamin-GFP and His2Av-mRFP for time lapse imaging, which was completed at various temperatures (11, 14, 18, 25, and 29 °C).

**Fig. 6:**
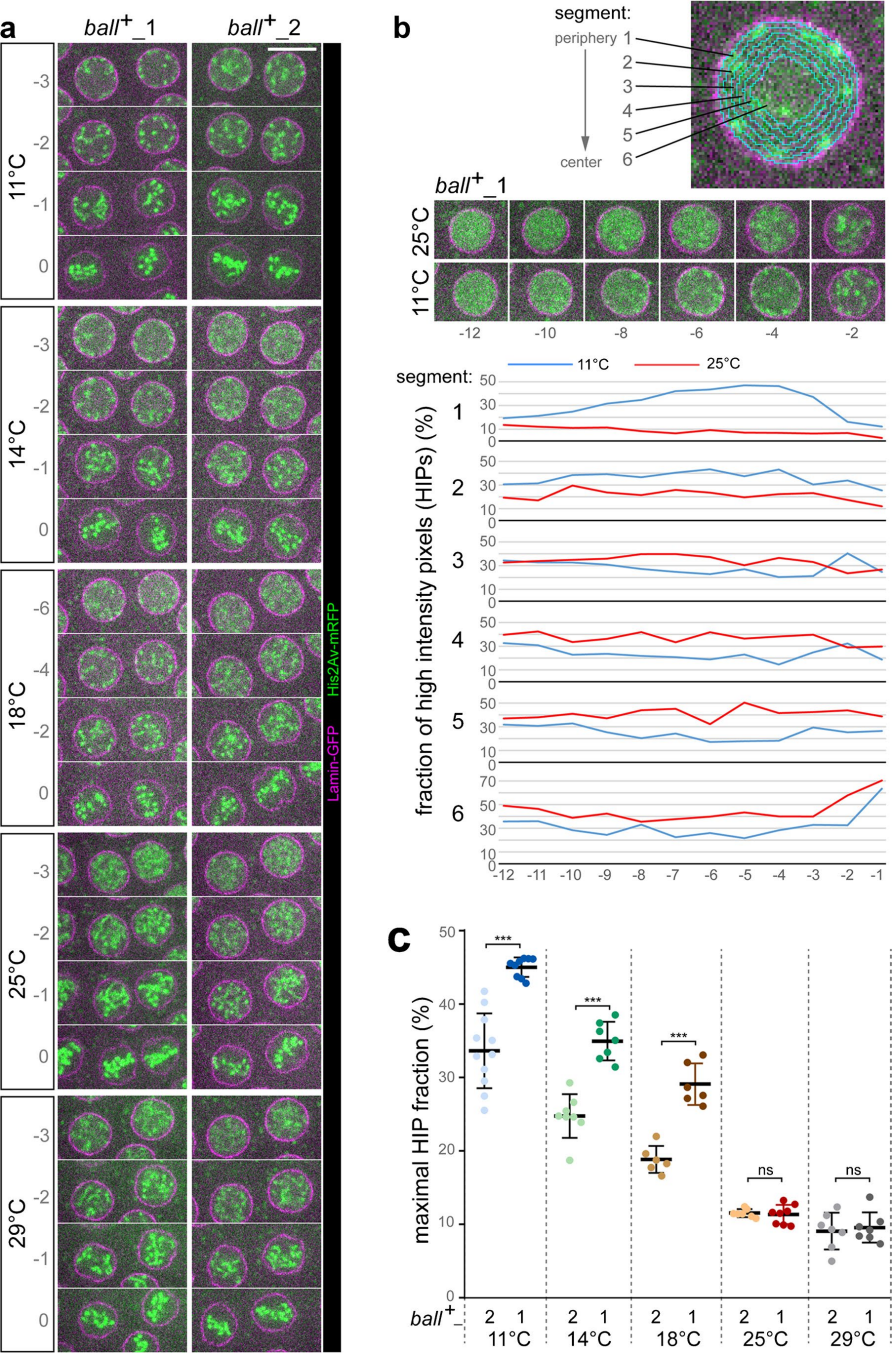
Delayed release of chromosomes from the nuclear periphery during prophase is enhanced by reduced *ball* function at low but not at high temperature. (**a**) Embryos expressing Lamin-GFP and His2Av-mRFP were analyzed by time-lapse imaging at the indicated temperatures. The number of *ball*^+^ gene copies in the mothers was either two (*ball*^+^_2) or only one (*ball*^+^_1). Time intervals between z-stack acquisitions were adjusted so that an identical number of z-stacks per cycle were acquired at the different analysis temperatures, except for a doubled acquisition rate in case of 18 °C. Single equatorial sections from the first z-stack that displayed the final metaphase plate of M12 (0) and from preceding z-stacks (− 1, − 2, − 3, …) are shown. Scale bar = 10 μm. (**b**) Quantitative analysis of the spatial pattern of chromosome condensation. The nuclear interior in equatorial sections was subdivided into six concentric segments (white lines) starting from the Lamin-GFP signals at the periphery. In each of the different segments, the fraction of high intensity pixels (HIP) displayed in the His2Av-mRFP channel was determined. HIPs arise from chromosome condensation and have a predominantly peripheral location before release of condensing chromosomes from the NE, as illustrated with equatorial sections from representative nuclei entering M 12 at either 25 or 11 °C. As in (A), the first z-stack displaying the final metaphase plate was designated as z-stack # = 0, and the preceding stacks were numbered accordingly (− 1, − 2, − 3, …). The numbers below the images indicate z-stack number, as well as those along the y-axis of the graphs that display HIP fractions over time in the different intranuclear segments. The increased HIP fraction in segment 1 at 11 °C reflects delayed chromosome release from the NE. (**c**) Comparison of the extent of peripheral chromosome condensation at the indicated temperatures in embryos with either normal or reduced *ball*.^+^ function. For each analyzed nucleus, the maximal HIP fraction value observed in segment 1 during the eight time points before metaphase plate formation was selected. Each dot represents the mean obtained by averaging these maximal values over all the nuclei analyzed from a given embryo. Mean ± s.d. obtained after averaging over all embryos is displayed as well. n nuclei per embryo ≥ 11. n embryos ≥ 6. *p* ≤ 0.001 *** (t test)

Time-lapse imaging of chromosome condensation at M12 onset in *ball*^+^_1 and *ball*^+^_2 embryos demonstrated that the extent of the delay in the release of condensing chromosomes from the nuclear periphery was highly dependent on both temperature and *ball*^+^ function (Fig. 6). Delayed chromosome release was not observed at 25 and 29 °C (Fig. 6a). Further confirmation that high temperatures do not result in a delayed release of condensing chromosomes from the NE was obtained by imaging at 30 °C (data not shown). In contrast, the delayed release was all the more apparent the lower the analysis temperature (Fig. 6a). Importantly, the delay observed at low temperatures appeared to be stronger in *ball*^+^_1 compared to *ball*^+^_2 embryos (Fig. 6a). Quantitative image analyses corroborated these conclusions concerning the compound effects of low temperature and reduced *ball*^+^ gene function on the release of condensing chromosomes from the nuclear periphery. For quantification, the spatial distribution of His2Av-mRFP signals above a threshold intensity was analyzed during entry into M12. These high intensity pixels (HIPs) arise from chromosome condensation. To determine the spatial distribution of the HIPs, nuclei and their equatorial z-sections were detected automatically in the time-lapse image data. The nuclear interior in the equatorial z-section was then subdivided into six concentric onion ring-like segments, and the fraction of HIPs located in these segments was determined (Fig. 6b). Peripheral chromosome condensation is indicated by high HIP fractions in peripheral segments. In contrast, a rapid release of chromosomes from the periphery during condensation is accompanied by high HIP fractions predominantly in central segments. Entry into M 12 at 11 °C was associated with a transient increase of the HIP fraction in the peripheral segments 1 and 2 (Fig. 6b). In contrast, at 25 °C, the increase in HIP fractions occurred preferentially in the internal segments 4–6 (Fig. 6b).

Since peripheral chromosome condensation is most clearly indicated by the HIP fraction in the segment 1, we used the maximal HIP fraction values observed in this most peripheral segment during the eight time points before metaphase plate formation for a statistical comparison (Fig. 6c). This comparison demonstrated that peripheral chromosome condensation during entry into M12 is promoted by low but not high temperature. Increased peripheral chromosome condensation reflects a delayed release from the NE. The lower the temperature, the more pronounced this phenotype was (Fig. 6c). Importantly, a reduction in the maternal *ball*^+^ gene dose clearly enhanced this phenotype at low but not at the presumed optimal or high temperature. The difference between *ball*^+^_1 and *ball*^+^_2 embryos was highly significant at 11 °C, 14 °C, and 18 °C (Fig. 6c). In contrast, there was no significant difference detectable at 25 °C and 29 °C (Fig. 6c). Therefore, it is concluded that *ball*^+^ function is clearly more important for a swift release of the condensing chromosomes from the nuclear periphery during mitosis at lower temperatures than at optimal and higher temperatures. The release of condensing chromosomes from the nuclear periphery proceeds with a reduced efficiency at lower temperatures, generating an increased sensitivity to reduced *ball*^+^ function.

To address whether temperature controls the level of Ball protein in syncytial embryos, we analyzed extracts prepared from *g-ball-EGFP* embryos after aging at different temperatures. Immunoblotting with anti-GFP did not reveal reduced levels of Ball-EGFP at low temperature (Online Resource 7, S4 Figure).

### Increased requirement for Ball/VRK protein kinase during wing imaginal disc cell proliferation at low temperature

**Fig. 7:**
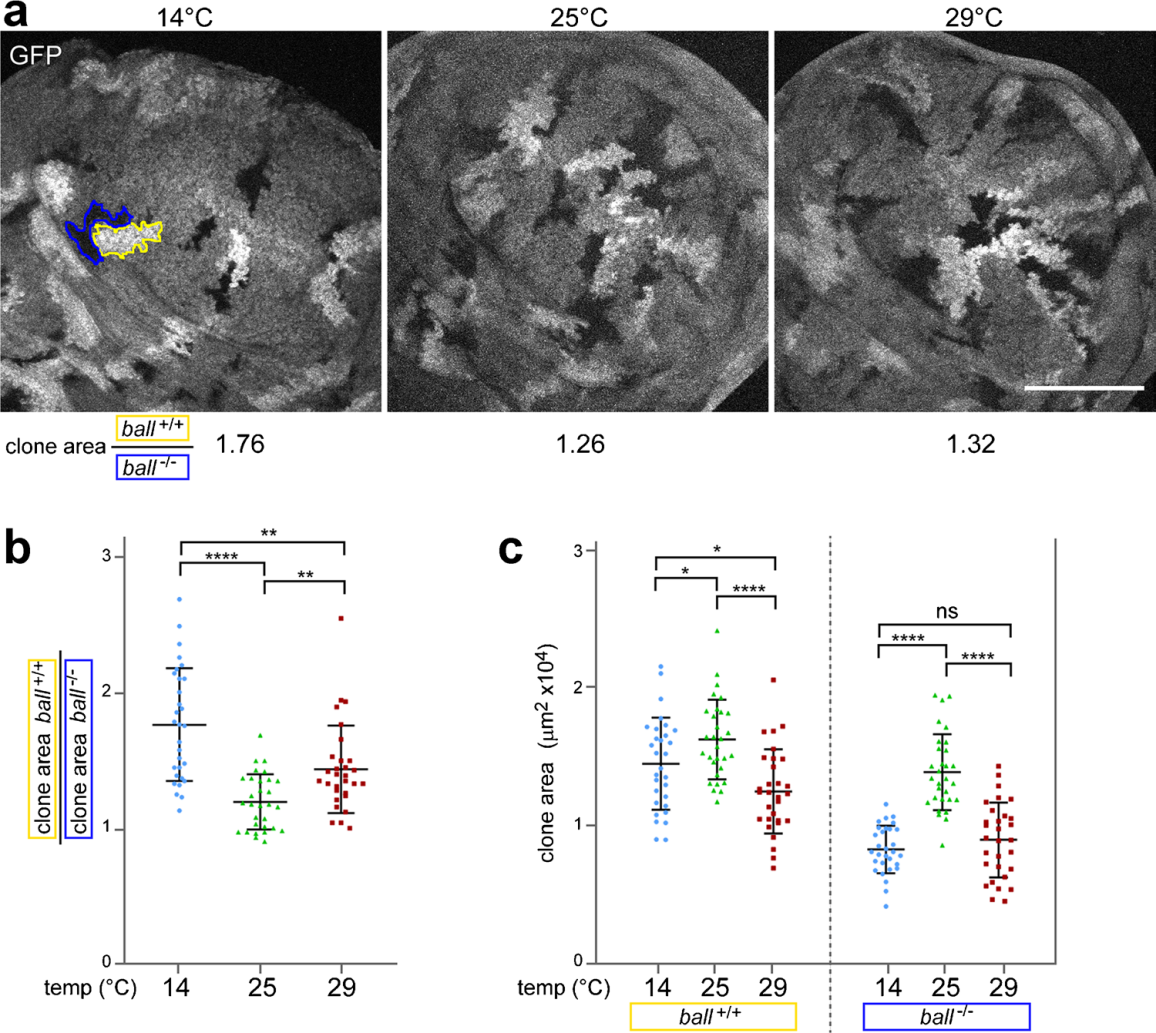
Increased *ball*^+^ requirement for mitotic proliferation at low temperature in wing imaginal discs. (**a–d**) Mitotic recombination was induced in *hs-FLP*; *FRT82B ball*^2^/*FRT82B UbiP-GFP* larvae around the 1st to 2nd instar transition followed by further aging at the indicated temperatures until third instar wandering stage. The area of *ball*^2^ clones (*ball*^−/−^) and of control clones (*ball*^+/+^) was determined after dissection and imaging of wing imaginal discs. (**a**) Wing pouch regions of representative imaginal discs are shown with a twin spot consisting of a *ball*^−/−^ and a *ball*^+/+^ clone delineated in blue and yellow, respectively. Numbers below the images indicate the observed ratio of the areas of all the *ball*^−/−^ and *ball*^+/+^ clones in the displayed discs. Scale bar = 100 µm. (**b–d**) Graphs displaying the ratios of the clone areas (b), the clone area of the *ball*^+/+^ clones (c), and of the *ball*^−/^.^−^ clones (d) at 14 °C, 25 °C, and 29 °C. Each point represents one wing imaginal disc. Mean values ± s.d. (*n* = 30) are indicated, as well as statistical significance (t test) with ns (not significant, *p* > 0.05), * (*p* ≤ 0.05), ** (*p* ≤ 0.01), and **** (*p* ≤ 0.0001)

To examine whether Ball/VRK is particularly important at low temperature not only in early embryos but also later in development, we performed clonal analyses during the larval stages, focusing on wing imaginal discs. Mitotic recombination was induced in early *ball*^2^ heterozygous larvae using FLP/FRT (Golic and Lindquist 1989), which can generate a pair of daughter cells, where one cell is homozygous for *ball*^2^ (*ball*^−/−^) and the other homozygous *ball*^+^ (*ball*^+/+^). This genetically mosaic cell pair is expanded into twin clones, if mitotic proliferation proceeds after clone induction. At 25 °C, *ball*-mutant clones in wing imaginal discs were shown to cover an area that was reduced in comparison to control clones (Cullen et al. 2005). To analyze temperature effects, we incubated larvae at either 14 °C, 25 °C, or 29 °C during development to the third instar wandering stage, after clone induction around the transition from the first to the second larval instar. After dissection and imaging of wing imaginal discs, we determined the areas covered by *ball*^+/+^ and *ball*^−/−^ clones, respectively. As expected (Cullen et al. 2005), after development at 25 °C, the mean ratio of these areas was 1.19 ± 0.21 (*n* = 30) (Fig. 7b), confirming that the growth of *ball*^−/−^ clones is impaired. Interestingly, at 14 °C, the ratio of the clone areas was significantly higher (1.76 ± 0.42, *n* = 30) (Fig. 7b). This increase at low temperature was caused by a more pronounced impairment of *ball*^−/−^ clone growth at 14 compared to 25 °C, as indicated by the size of the clone areas (Fig. 7c). While the mean area of the *ball*^+/+^ clones was only slightly reduced at 14 compared to 25 °C, that of the *ball*^−/−^ clones was far more strongly reduced (Fig. 7c). Similar to the results obtained at 14 °C, the ratio of the clone areas was also higher at 29 °C (1.43 ± 0.32, *n* = 30) in comparison to 25 °C (Fig. 7b). However, the increase of the clone area ratio at 29 °C was significantly lower than at 14 °C (Fig. 7b), because it was not primarily the growth of the *ball*^−/−^ clones but also that of the *ball*^+/+^ clones, which was compromised at 29 compared to 25 °C (Fig. 7c). In conclusion, the clonal analyses indicated that *ball*^+^ function is most important for cell proliferation at 14 °C in comparison to 25 °C and 29 °C.

## Discussion

The actual impact of temperature on the myriad of cellular processes is still poorly understood. Here, we have applied genome-wide RNAi screening for an identification of genes with functions that appear to be of temperature-dependent importance for *Drosophila* S2R + cells. Based on a comparison of knockdown effects on cell number and cell cycle profile at 17 and 27 °C, respectively, candidate genes with a temperature-dependent physiological significance were identified. The quality of our screen data was evaluated and confirmed by independent validation experiments with selected hits. Moreover, our initial characterization of one of the screen hits in the organism indicated that the corresponding gene, *ballchen* (*ball*), which codes for the *Drosophila* homolog of the VRK protein kinase, is more critical at low temperature not only in cell culture but also during organismal development.

We are not aware of any previous genome-wide screens for genes of particular significance at low temperature in *D. melanogaster* or in any other animal model organisms. Such screens have been realized with the unicellular species *Bacillus subtilis* and *Saccharomyces cerevisiae* (Abe and Minegishi 2008; Jesus Ferreira et al. 2001; Koo et al. 2017). In these unicellular organisms, the role of non-essential gene for growth at low temperature was analyzed with gene deletion libraries generated comprehensively using homologous recombination or near comprehensively by transposon tagging mutagenesis. The fact that large-scale screens with multicellular organisms are technically far more challenging than with unicellular organisms probably presumably explains why this approach has not been applied so far in the field of animal cold acclimation. However, RNA interference, or in principle also CRISPR/cas, with cultured animal cells provides a technically feasible avenue to large-scale screening, as demonstrated here with *D. melanogaster* cells.

RNAi screening has well-known limitations (Heigwer et al. 2018). In particular, off-target effects, although minimized by the computational design of the specificity-optimized dsRNA library (Horn et al. 2010), might still unravel and cause occasional false-positive results. Moreover, false-negative results can arise because of incomplete knockdown. Even if a target mRNA is effectively eliminated by a given dsRNA, products translated before dsRNA addition may survive in particular in case of stable proteins and provide sufficient function, thus masking knockdown effects. In our screen, the relevance of incomplete knockdown is accentuated because RNAi treatment was for only 3 days in the 27 °C replicates. This duration is still sufficient to permit at least two doublings of the cell population in mock-treated controls. In case of the 17 °C replicates, 10 days were adopted for the RNAi treatment, since this allowed a comparable control cell population increase according to preliminary tests. During the screen, cell proliferation turned out to be lower at 17 compared to 27 °C. However, this is unlikely to have had a profound impact on our hit lists, as they were generated by comparing B-scores obtained at 17 and 27 °C, respectively, and if anything, it makes the genes that we have identified as of particular importance at 17 °C even stronger candidates. Clearly, it is possible that we might have incorrectly attributed temperature-dependent significance to a particular gene because the RNAi-mediated elimination of the target mRNA or the protein stability of the final gene product was temperature-dependent in the screen. Yet, our hit identification by comparing 17 versus 27 °C B scores should largely avoid such errors except in cases of a gene-specific temperature dependency of the knockdown efficiency, which is likely rare. Moreover, judging from our 222 positive control wells with dsRNA targeting the anti-apoptotic gene *Diap1*, knockdown efficiency at 17 and 27 °C was similar (Online Resource 8, S1 text).

Analysis of cell numbers permits an effective identification of, in particular, those genes that result in cell death when knocked down. If knockdown merely halts cell cycle progression without triggering cell death, cell numbers are not a very sensitive indicator, especially when the extent of proliferation of control cells is limited during the RNAi treatment period. Therefore, beyond cell numbers, we have also used cell cycle profiles as read-out. Cell cycle profiles can be extracted readily from the images acquired during our automated microscopic screening. The cell cycle profile corresponds to the histogram obtained after quantification of the individual nuclear DNA signal intensities in a given well. After knockdown of the known cell cycle regulators *CycE* and *stg*/*cdc25*, for example, the expected abnormal cell cycle profiles were clearly detected, indicating a cell cycle arrest during G1 and G2, respectively. Importantly, the cell cycle profiles observed in negative control wells were largely identical at 17 and 27 °C. The cell cycle profiles resulting from knockdown of known cell cycle regulators like *CycE, stg*/*cdc25*, and *Cdk1* were also very similar at 17 and 27 °C. Interestingly, however, knockdown of some genes, including *Pvr* and *Dsor1*, resulted in very distinct cell cycle profiles at 17 and 27 °C, respectively. While the overlap between the screen hit lists with genes being more important at 17 than at 27 °C for either normal cell proliferation or a normal cell cycle profile was substantial, as expected, analysis of the cell cycle profile also identified candidate genes that were not revealed by cell count abnormalities.

We emphasize that we have not applied highly restrictive criteria during the generation of our hit lists with genes of temperature-dependent significance for S2R + cell proliferation. These lists were prepared primarily to support a selection of candidate genes for further functional characterization ideally in the organism. An overall comparison of our RNAi screen with that of Boutros et al. (2004) (Boutros et al. 2004), in which a luciferase-based assay for ATP-levels was used as a proxy for cells numbers after knockdown at 24 °C (with Kc_167_ cells and two replicates), illuminates that our hit selection was of limited stringency. In the latter screen, 438 genes were scored as “essential” (z-score > 3), while in our screen 1931 genes, i.e., fourfold more, were associated with “unusually low cell counts” after RNAi at 27 °C (B-score ≤  − 2). Further validation of candidates present on our hit lists is also important because the two replicates per temperature analyzed in our screen, while informing about reproducibility, are hardly adequate for a statistically robust identification of genes associated with more pronounced knockdown effects at 17 compared to 27 °C. A systems biological analysis aimed at a comprehensive identification of pathways crucial for acclimation to low temperature was thus not attempted. However, we point out that our RNAi screen data not only highlight candidate genes of temperature-dependent significance, but also contains additional data for an identification of cell proliferation and cell cycle genes of importance at both temperatures that could also be integrated with previously published RNAi screens (Bjorklund et al. 2006; Boutros et al. 2004). Moreover, the acquired images can be used for an analysis of additional cellular features revealed by the microtubule and DNA staining. An additional caveat concerns potential differential effects on culture medium contents by incubation for 3 days at 27 °C compared to 10 days at 17 °C.

Beyond limitations discussed above, evidence demonstrates that our screen data is of high technical quality. In our validation experiments, the effects observed in the screen were confirmed for 87.5% of the re-tested hits when using independent dsRNA preparations of identical sequences for screen and validation. RNAi screen data quality was also highlighted by the enrichment of Gene Ontology (GO) terms associated with the gene list derived from dsRNA amplicons linked to unusually high cell numbers at both temperatures (S2 Table, l2_both_strong). While some attenuation of cell proliferation is probably resulting from knockdown of very many genes, the opposite, accelerated cell proliferation due to a gene knockdown, is arguably much less likely. Accelerated cell proliferation might arise after depletion of negative regulators of cell proliferation, or alternatively it might simply reflect assay noise rather than real knockdown effects. In the latter case, a statistically significant enrichment of meaningful GO terms is not to be expected. However, the GO terms “negative regulation of cell cycle” (*p* = 2.272163e-2) and “negative regulation of mitotic cell cycle” (*p* = 6.035725e-4) were indeed significantly enriched by the high cell number gene list, which included all the well-characterized negative cell cycle regulators Rbf (Retinoblastoma protein homolog), Dacapo (CIP/KIP-type Cdk inhibitor), Myt1 (protein kinase inhibiting Cdk1), Wee1 (protein kinase inhibiting Cdk1), Trbl (antagonizing Stg/Cdc25 phosphatase), Rad9 (DNA damage checkpoint), Grp/Chk1 (DNA damage checkpoint), and Bub1 (spindle assembly checkpoint). Furthermore, the statistically significant enrichment of certain GO terms by the lists l17weak and l27weak was distinct. For example, a conspicuous enrichment of terms linked to RNA splicing was specific for the l17weak genes. Interestingly, extensive alterations of splice patterns and poly A site selection were reported to accompany temperature change within the readily tolerated range in adult flies and S2R + cell (Jakšić and Schlötterer 2016).

The analyses of Bai et al. (2021) are also of interest in the context of our identification of *Pvr* and *Dsor1* as genes that are more important for a normal cell cycle profile at 17 compared to 27 °C. Based on expression profiling, assays for transposase-accessible chromatin using sequencing (ATAC-Seq) and a novel enhancer assay, Bai et al. (2021) have identified a *cis*-regulatory element from the *pastrel* (*pst*) gene that mediates transcriptional upregulation in S2R + cells at low temperature. Interestingly, the activity of this *pst*_E1 enhancer was found to depend on the function of Pvr, a receptor tyrosine kinase, which signals through Dsor1 (Duchek et al. 2001; Ragab et al. 2011). The mRNA levels of genes coding for the Pvr ligands, Pvf2 and Pvf3, were also found to be upregulated in S2R + cells at low temperature (Bai et al. 2021). While clearly required for cell proliferation also at the optimal temperature, Pvr signaling appears of particular significance at lower temperatures, and a future detailed analysis of activation and function of this pathway at low temperature should be of interest.

For an initial evaluation of temperature dependence not only in cultured cells but also in the organism, we have selected one strong screen hit, the gene *ball*, which codes for the *Drosophila* Ball/VRK protein kinase homolog. Both in the genome-wide RNAi screen and in the validation experiment, *ball* was found to be more important for S2R + cell proliferation at 17 compared to 27 °C. Similarly, our clonal analyses in wing imaginal discs indicated that cell proliferation during larval development is reduced more strongly at low temperature in clones lacking *ball* function. Moreover, our analysis of chromosome release from the NE at the onset of M phase, a process known to be promoted in metazoans by Ball/VRK activity (Gorjánácz et al. 2007; Nichols et al. 2006; Segura-Totten and Wilson 2004), clearly revealed an interaction between temperature and *ball* function as well. By time-lapse imaging, chromosome release from the NE during entry into the syncytial blastoderm division M12 in early embryos was observed to occur with a delay at low temperatures. Moreover, this delay was increased in embryos with reduced maternal *ball* function, but only at low temperatures.

Recent analyses have independently demonstrated that low temperature induces a delayed release of condensing chromosomes from the NE at the onset of syncytial blastoderm divisions (Falahati et al. 2021). Based on time-lapse imaging of Cyclin B (CycB)-GFP and a biosensor reporting the activity ratio of Cdk1 and phosphatase 1, low temperature was shown to delay nuclear CycB-GFP accumulation and Cdk1 activation to the level sufficient for triggering NEBD and thus prometaphase onset (Falahati et al. 2021). While the low temperature effect on CycB-Cdk1 activation dynamics remains to be clarified at the molecular level, translocation of CycB into the nucleus might well be the temperature-sensitive step. Low temperature was shown previously to cause a strong reduction in the level of O-GlcNAc (Radermacher et al. 2014), a posttranslational modification which occurs most prominently on protein subunits of the nuclear pore (Davis and Blobel 1987; Hanover et al. 1987; Holt et al. 1987; Onischenko et al. 2004; Snow et al. 1987), where it increases pore permeability (Labokha et al. 2013; Yoo and Mitchison 2021). Additional research will be required to evaluate the potential link between O-GlcNAc and CycB-Cdk1 activation. Similarly, the regulation of Ball/VRK activity at the onset of M phase, possibly by CycB-Cdk1, is not yet understood. However, the detailed characterization of the effects of temperature on progression through syncytial blastoderm mitoses (Falahati et al. 2021) provides a clear demonstration that temperature change has differential effects on distinct cellular processes. Beyond disproportional lengthening of prophase in particular, metaphase was also found to be extended at low temperature compared to interphase, prometaphase, anaphase, and telophase. Although low-temperature effects on microtubule dynamics (Li and Moore 2020; Myachina et al. 2017) might contribute to the non-uniform temporal scaling of cell cycle phases, the tremendous complexity of cellular processes leaves room for many potential targets with pronounced low-temperature sensitivity.

The genome-wide RNAi screen with S2R + cells that we have completed should provide a helpful starting point for the informed selection of candidate genes for a further characterization of their differential temperature-dependent physiological significance in cells and organism. Their future functional characterization will hopefully close some of the many gaps in our mechanistic understanding of cellular acclimation to low temperature within the readily tolerated range.

## Materials and methods

### Cell culture

S2R + cells, originally derived from late stage embryos (Yanagawa et al. 1998), were cultured in Schneider’s cell culture medium (Gibco, #21,720). The medium was supplemented with 10% fetal bovine serum (Gibco, #10,500–064) and 1% penicillin–streptomycin (Gibco, #15,140).

### Genome-wide RNAi screen

A detailed description of the procedure used for completion of the genome-wide RNAi screen is provided as supplementary information (Online Resource 8, S1 Text). In brief, the dsRNA samples of the HD2 *Drosophila* genome-wide RNAi library (Horn et al. 2010) were aliquoted for the generation of four replicates (250 ng dsRNA per well). Each 384-well plate contained several negative control wells (no dsRNA) and positive control wells (*Diap1* dsRNA). After addition of S2R + cells (1.3 × 10^4^ cells/well), two replicates were incubated at 17 °C for 10 days and two replicates at 27 °C for 3 days. Thereafter, cells were fixed and stained with Hoechst 33,258 (0.7 µg/ml) and fluorescein isothiocyanate (FITC)-conjugated mouse monoclonal antibody DM1A anti-α-Tubulin (Sigma, #F2168) (1:1429). The microplates were imaged with an ImageXpress Micro High-Content Imaging System (Molecular Devices). A 4 × /0.2 air objective was used for imaging of the blue channel. In addition, the blue and green channels were imaged from each well by acquiring single optical sections from nine partially overlapping regions with a 20 × /0.45 air objective.

The 4 × images of the DNA staining signals were used for an initial identification of screen hits. These images were processed into whole-plate images. The signal intensity detected in the region of a particular well indicated, to a rough approximation, the total number of cells present in that well. Visual comparison of whole plate images of the 17 and 27 °C replicates allowed an identification of wells, in which cell number was low specifically at the lower temperature.

The 20 × images were processed before analysis with CellProfiler pipelines (McQuin et al. 2018). The pipelines provided a cell count for each well (Online Resource 1, S1 Table), as well as for each nucleus within a well the integrated DNA staining signal intensity. To create hit lists based on the cell counts (S2 Table), the B-score (Brideau et al. 2003) of the cell count of each well (Online Resource 1, S1 Table) was calculated with a Python script. FlyBase annotations (release 5.45) were used to assign dsRNA amplicons to genes. For identification of screen hits based on the cell cycle profile, a histogram displaying the frequency distribution of the nuclear DNA contents in a given well was generated for each well with a Python script. For a parametric description of the cell cycle profile, a cell population model was fitted to the DNA signal intensity histogram. The basic assumption of this model was that the cell population in each well is comprised of three distinct Gaussian sub-populations P1, P2, and P3. In unperturbed cells, the three sub-populations correspond essentially to the G1 cells, to the G2/M cells, and to abnormal hyperploid cells, respectively. The frequency of cells in these three populations was calculated (P1, P2, P3) (Online Resource 1, S1 Table). Robust Z-scores (Birmingham et al. 2009) were calculated for P1, P2, P3, and for the P1/P2 ratio. The robust Z-scores (Online Resource 1, S1 Table) were then used for the generation of lists (S3 Table).

The hit lists were analyzed for enrichment of gene ontology (GO) terms using FlyMine (https://www.flymine.org/flymine) (Lyne et al. 2007) with Holm-Bonferroni correction (maximal *p*-value set to 0.05). In addition, with the average B-scores of the cell counts at 17 and 27 °C, respectively, an analysis for functional enrichment was also performed using the STRING database and its function “Protein(Gene) with Values/Ranks” (Szklarczyk et al. 2021).

### Validation of screen hits based on cell count analysis

For validation of RNAi screen hits that appeared important for cell proliferation preferentially at the low temperature, independently generated dsRNA amplicons were used for knockdown and analysis in experiments analogous to the screen. DNA fragments for production of dsRNA by in vitro transcription were amplified from S2R + cell genomic DNA by PCR using the primers described in Online Resource 9 (S4 Table), which also specifies the amplicon identifiers as in the GenomeRNAi database v17 (http://www.genomernai.org.) (Schmidt et al. 2013). DNA fragments were purified from an agarose gel using a gel extraction kit (QIAGEN, #28,706) and transcribed using Ambion T7 Megascript Kit (Invitrogen, #AM1334). The MEGAclear Transcription Clean-Up Kit (Invitrogen, #AM1908) was used for cleaning of the dsRNAs. NanoDrop (Thermo Scientific, ND-ONEC-W) was used to determine their concentration. Experiments were performed in duplicates or triplicates, except for *Rheb*, *Cdk12*, and *fwd*, where only one well was analyzed. As a negative control, dsRNA targeting *lacZ* was included. For validation of hits based on the analysis of overall DNA signal intensities, experiments were performed in 96-well plates using the pipetting robot Biomek EL406 and imaging with an automatized microscope Micro XL (Molecular devices) with a 20 × /0.4 air objective. For validation of hits based on the CellProfiler analyses, experiments were set up manually in 24-well plates and imaged using a microscope Zeiss CellObserver HS with 20 × /0.5 objective. The final concentrations of cells, dsRNA, and reagents used during fixation and staining in the validation experiments were comparable to those used in the screen. Incubation with dsRNA in serum-free medium was for 1 h. Fixation and staining were shortened to 15 and 60 min, respectively. A modified CellProfiler pipeline was used for data extraction. Nuclei were detected as the primary objects based on the DNA staining (blue channel) and counted. The cells were detected as the secondary objects, based on α-Tub signals (green channel) around the primary objects. A threshold for signal intensity in the green channel was applied in some experiments for an exclusion of cell debris. The pipeline parameters were adjusted to achieve a most accurate determination of cell counts based on visual inspection of the analyzed images. The cell counts observed in replicates treated with a given dsRNA preparation at 17 °C were averaged and normalized to the cell counts observed with the negative control at this temperature. Analogously, cell counts observed at 27 °C with the given dsRNA preparation were averaged and normalized. Finally, the normalized cell counts were used for the calculation of the ratio of cell counts at 17 and 27 °C. The standard deviation of this cell count ratio was calculated using error propagation.

### Validation of screen hits based on cell cycle profile analysis

Validation of screen hits associated with abnormal cell cycle profiles especially at low temperature was again done by analyzing the effects of independently generated dsRNAs after treatment of S2R + cells as in the screen. The dsRNA was generated as described above. For negative controls, cells were treated with either no dsRNA or dsRNA targeting the *lacZ* gene. The validation experiments were performed in 6-well plates. In contrast to the primary screen, the cell cycle profile was determined by flow cytometry rather than by quantitative imaging microscopy. Before flow cytometry, S2R + cells were detached with trypsin–EDTA. Cells were sedimented, washed with phosphate buffered saline (PBS) (137 mM NaCl, 2.7 mM KCl, 1.47 mM KH_2_PO_4_, pH 7.4) and resuspended in 1 ml of PBS. The Live/Dead Fixable Violet Dead Cell Stain (ThermoFisher, #L34955) was added to a final concentration of 1:1000 in PBS. After incubation for 30 min at RT in the dark, cells were washed with PBS and resuspended in 0.5 ml of PBS. For fixation, 5 ml of 95% ethanol was added. Fixed cells were stored at 4 °C for maximally 2 weeks before analysis by flow cytometry. On the day before flow cytometric analysis, cells were resuspended in 1 ml PBS. For degradation of RNA and staining of DNA, 25 µl of RNase A stock solution (1 mg/ml) and 25 µl of propidium iodide (1 mg/ml, Fluka, #81,845) stock solution were added. After incubation overnight at 4 °C, cells were filtered with a Cell-Strainer (Falcon, #352,235) and kept on ice until flow cytometric analysis with a BD LSR II Fortessa or a BD FACSymphony instrument at the Cytometry Facility (University of Zurich, Irchel Campus). Live/dead stain was excited with a 405-nm laser, and emission was detected with either 450-/50-nm bandpass filter (BD LSR II Fortessa) or with a 425-nm longpass and a 450-/50-nm bandpass filters (BD FACSymphony). Propidium iodide was excited with a 561-nm laser, and emission was detected with a 685-nm longpass and a 710/50-nm bandpass filter (BD LSR II Fortessa) or with a 600-nm longpass and a 610-/20-nm bandpass filter (BD FACSymphony). For each sample, at least 50,000 events were recorded. The data analysis was performed in FlowJo (Becton Dickinson, Franklin Lakes, NJ, USA). Exclusion of debris was achieved by gating based on forward scatter (FSC) and side scatter (SSC) values. Exclusion of dead cells was achieved based on gating based on the live/dead staining signals. Gates were set on the negative controls and kept for all samples of an experiment. Cell cycle histogram parameters were calculated with the Watson model, using ranges for G1 and G2 peaks constrained based on profile of the negative controls.

### Fly strains and crosses

*D. melanogaster* was cultured using standard fly food. Lines with the *ball* mutant alleles E107 and E24 (Cullen et al. 2005) were kindly provided by Hiroyuki Ohkura (Wellcome Trust Centre for Cell Biology, University of Edinburgh, Edinburgh, UK). *ball*^*2*^ as well as the line with the transgene *g-ball2.1* and *g-ball-GFP* (Herzig et al. 2014) were kindly provided by Alf Herzig (Max Planck Institute for Infection Biology, Berlin, Germany). Expression of this transgene is controlled by the *ball* cis-regulatory region, as also in case of *g-ball-GFP* (Vienna Drosophila Resource Center, #318,678). For clonal analyses, we generated a recombinant chromosome *P{ry[*+ *t7.2]* = *neoFRT}82B, e, ball*^*2*^ free of second site lethal mutations. Absence of second site lethal mutations was confirmed by the demonstration that the developmental lethality of homozygous *P{ry[*+ *t7.2]* = *neoFRT}82B, e, ball*^*2*^ flies was completely suppressed by the presence of the *g-ball-GFP* insertion on chromosome 2. Lines with *P{hsFLP}12* (BDSC #1219) and *P{ry[*+ *t7.2]* = *neoFRT}82B, P{w[*+ *mC]* = *Ubi-GFP.D}* (BDSC #5188) were obtained from the Bloomington Drosophila Stock Center (BDSC). The transgenes *P{w[*+ *mC]* = *His2Av-mRFP1}II.2* (Schuh et al. 2007) and *P{PTTGB}G262* (Morin et al. 2001), which expresses GFP-tagged Lamin, were recombined for time-lapse analysis with early embryos. For these analyses, an additional recombinant chromosome *P{ry[*+ *t7.2]* = *neoFRT}82B, e, ball*^*2*^, *P{g-ball}2.1* was used. *Oregon-R* or *w* flies were used as wild-type flies.

Standard crossing was applied for the generation of the female flies, from which embryos were collected and analyzed by time-lapse imaging. The genotype of these female flies was w*; *P{w[*+ *mC]* = *His2Av-mRFP1}II.2*, *P{PTTGB}G262/* + ; *P{ry[*+ *t7.2]* = *neoFRT}82B, e, ball*^*2*^/ + or w*; *P{w[*+ *mC]* = *His2Av-mRFP1}II.2*, *P{PTTGB}G262/* + ; *P{ry[*+ *t7.2]* = *neoFRT}82B, e, ball*^*2*^, *P{g-ball}2.1*/ + . Before egg collection, these females were mated with *w* males.

For the generation of twin spot clones, we crossed *y* w*, P{hsFLP}12*;; *P{ry[*+ *t7.2]* = *neoFRT}82B, P{w[*+ *mC]* = *Ubi-GFP.D}* virgins with *P{ry[*+ *t7.2]* = *neoFRT}82B, e, ball*^*2*^/ *TM6B, Tb, Hu* males. Embryos were collected in fresh tubes during 2 h at 25 °C and further aged for 48 h at 25 °C. To apply a heat shock for *hsFLP* expression, vials were incubated for 18 min in a 37 °C water bath. After the heat shock, vials were returned to 25 °C for 1 h before transfer to either 14 °C, 25 °C, or 29 °C. Non-*Tb* larvae during third instar wandering stage were used for isolation of wing discs.

### Analysis of twin spot clones in wing imaginal discs

Wing imaginal discs were dissected from third instar wandering stage larvae in PBS. Wing discs were fixed at room temperature for 20 min with 4% formaldehyde in PBS. After washing three times for 5 min with PBS, discs were stained with Hoechst 33,258 (1 µg/ml) for 10 min. After an additional three washes with PBS for 5 min each, discs were mounted in Vectashield Antifade Mounting Medium (Vector Laboratories, #H-1000). An Olympus FluoView 1000 laser-scanning confocal microscope was used for imaging with a 40 × /1.3 oil objective. Ten optical sections with a spacing of 1 µm were acquired with a Kalman filter setting of three.

ImageJ was used for quantification of the clone areas. The z-slice that contained a maximal number of nuclei in the flat wing pouch region was identified. This and the two flanking slices above and below were used for the generation of a maximum intensity projection along the z-axis. The z-projection was blurred with a Gaussian filter (2 pixel diameter). The regions with very bright GFP signals (clones with cells homozygous for the chromosome 3 arm with the GFP marker gene) were manually selected. Similarly, the regions with only background signals in the green channel (clones with cells homozygous for the chromosome 3 arm with *ball*^2^ and without the GFP marker gene) were also manually delineated, although using a blurred minimum intensity projection of the same three z sections. For each wing disc, we determined the sum of the selected areas for the two types of clones.

### Time-lapse imaging and analysis with early embryos

Before egg collection, parent flies of the appropriate genotype were combined and pre-mated for at least 3 days at 25 °C. Eggs were collected on yeasted apple juice agar plates during 45 min at 25 °C, followed by aging for another 30 min at 25 °C. Embryos were dechorionated with sodium hypochlorite during 2 min at room temperature. After extensive washing with water, the dechorionated embryos were aligned along the edge of on an agar block and picked up with a cover slip coated with glue. Embryos on the coverslip were covered with pre-equilibrated mineral oil (Sigma Diagnostic Inc, St. Louis, USA, #021K6096) within in an imaging room maintained at the analysis temperature (11, 14, 18, 25, or 29 °C). A gas-permeable membrane (BioFolie 25, Sartorius, Goettingen, Germany) was applied on top of the mineral oil. The coverslip was then taped to a custom-made aluminum frame and mounted on the stage of a spinning disc confocal microscope with a 100 × /1.4 oil objective (VisiScope Spinning Disk Confocal System with a CSU X1 Yokagawa Spinning Disk and a Photometrics “evolve” EM 512 digital EMCCD camera equipped for red/green dual channel fluorescence observation from Visitron on an inverted Olympus IX83 microscope). The average duration between the transfer of the embryos to the analysis temperature and the start of imaging was 40 min (11, 14, and 18 °C), 30 min (25 °C), and 15 min (29 °C). However, variation in these durations was not found to have an obvious effect on the analyzed chromosome detachment phenotype at the onset of mitosis.

Image stacks of around 25 optical sections with 1-µm spacing were acquired at time intervals that were adjusted in length so that the number of stacks per cell cycle was comparable at the different analysis temperatures, except in case of 18 °C. The duration of the time intervals was 110 s at 11 °C, 65 s at 14 °C, and 25 s at both 25 and 29 °C. In case of 18 °C, an interval of 20 s was used, resulting in an approximate doubling of the number of stacks per cell cycle compared to 25 °C. All other acquisition parameters including laser power were kept identical at the different analysis temperatures. Imaging was started at anaphase/telophase of mitosis (M) 11 until anaphase/telophase of M12.

For quantitative analysis of the spatial pattern of chromosome condensation during entry into M phase, time-lapse data was analyzed with a custom-made ImageJ plugin. In a first step, the plugin detected individual nuclei as objects based on the His2Av-mRFP signal. Nuclei too close to the border of the image stacks or too close to another nucleus were excluded from the analysis. The selected nuclei were then tracked over time. A separate hyperstack was generated for each individual nucleus and pre-processed manually before further automated analysis. The time points covering period of interest from late interphase/early prophase of cycle 12 until prometaphase of cycle 12 were identified and extracted. This resulted in retention of 8 consecutive time points in case of the analyses at 11, 14, 25, and 29 °C and 16 time points in case of 18 °C. Moreover, excess z-sections above and below the nucleus were discarded. A second plugin was used for automatic determination of the nuclear periphery and the equatorial z-section for each nucleus at each time point based on the Lamin-GFP signal. The few nuclei, for which the automatic detection of the nuclear periphery was inaccurate, were excluded from further analysis. The equatorial z-sections were used for the quantification of spatial pattern of chromosome condensation at the start of mitosis. The circular disc with the nuclear interior bounded by the Lamin-GFP signal was segmented with six concentric rings by increasing the distance of the ring from the lamin-GFP signal stepwise by two pixels per step. Moreover, a histogram of all the pixel intensities within the circular disc with the nuclear interior was made for the His2Av-mRFP channel. A threshold pixel intensity was selected so that 30% of all the intranuclear pixels had a His2Av-mRFP signal intensity above the chosen threshold value. Intranuclear pixels with His2Av-mRFP signal intensity above the chosen threshold are the “high intensity pixels (HIP).” These HIPs are primarily associated with the condensing chromosomes during mitosis. The plugin calculated for each concentric nuclear onion ring segment (convex layer), the fraction of HIPs in the given segment. A HIP enrichment in the onion ring segment closest to the NE indicates peripheral chromosome condensation. Conversely, a HIP enrichment in the most central segment indicates internal chromosome condensation. To visualize the pattern of chromatin condensation over time for a given genotype at a certain temperature, the HIP fraction in the most peripheral intranuclear segment for all the analyzed nuclei of an embryo was averaged at each of the consecutive time points. Thereafter, these embryo averages were averaged over all embryos of the same genotype and temperature. Analogously, the average HIP fraction was determined for the most central intranuclear segment. The average HIP fraction with standard deviations of the most peripheral and most central segment was then graphed over time. For a quantitative comparison of the extent of chromosome condensation along the nuclear envelope at different temperatures and for different genotypes, a first step consisted in the determination of the maximal HIP fraction value within the most peripheral segment of a given nucleus within the analyzed time window. These maximal values were then averaged over all the nuclei of an embryo and thereafter over all embryos of the same genotype and temperature. The resulting average is correlated with the extent of peripheral chromosome condensation.

For figure preparation, still frames were selected, assembled, and adjusted (contrast and brightness) with ImageJ.

### Immunoblotting

Embryos were collected for 20 min on apple agar plate at 25 °C. After aging embryos at 25 °C for 45 min, 30 min, and 30 min, they were shifted to 14 °C, 25 °C, or 29 °C, respectively. Temperature shift to these assay temperatures was achieved by floating the collection plates on a water bath in an incubator pre-equilibrated to the desired temperature. Incubation at the analysis temperature was for 40 min at 14 °C, 30 min at 25 °C, and 30 min at 29 °C. After dechorionization with 3% sodium hypochlorite and rinsing with tap water, the embryos were homogenized in 3 × Laemmli buffer (62.5 mM Tris–Cl, 10% glycerol, 5% β-mercaptoethanol, 3% SDS, 0.01% Bromophenol Blue) on ice by grinding with a pestle in an Eppendorf tube, followed by heating for 4 min at 95 °C. Extracts were cleared by centrifugation at 4 °C, flash frozen using liquid nitrogen, and stored at − 80 °C until analysis by immunoblotting. Concentration of proteins in each sample extract was measured using Pierce™ 660 nm Protein Assay Reagent (ThermoFisher Scientific, cat #22,660). For immunoblotting, 20 µg of total proteins were loaded per lane and resolved by standard SDS-PAGE on a BioRAD mini-gel apparatus (2 h, 100 V) and transferred to nitrocellulose membrane (Amersham™ Protran® 0.45-μm NC) by tank blotting (1 h 15 min, RT, 100 V). Membranes were incubated with blocking solution (1 × PBS containing 5% w/v dry milk and 0.1% Tween-20) for 45 min. Rabbit anti-GFP antibodies (Torrey Pines Biolabs, TP401) were diluted 1:2000 and applied overnight at 4 °C. Peroxidase-conjugated goat anti-rabbit antibodies (Jackson ImmunoResearch) were used as secondary antibodies at a dilution of 1:1000 and applied for 1.5 h at RT. Mouse anti-Lamin antibodies (DSHB, ADL67.10 Dm0) were diluted 1: 200 and applied overnight at 4 °C. Peroxidase-conjugated goat anti-mouse antibodies (Jackson ImmunoResearch) were used as secondary antibodies at a dilution of 1:1000 and applied for 1.5 h at RT. Signals were detected by chemiluminescence (WesternBright ECL, advansta R-03031-D25), and images were acquired using an Amersham Imager 600.

## Supplementary Information

Below is the link to the electronic supplementary material.

Supplementary file1 S1 Table. RNAi screen data generated by image analysis. Data obtained by image analysis is given for each well analyzed in the genome-wide RNAi screen. Sixty 384-well plates (23040 wells) were imaged in each of the four replicates (replicate_17°C_1, replicate_17°C_2, replicate_27°C_1, replicate_27°C_2). The information listed for each well includes the intended target gene, the number of nuclei in the well, B-scores of the number of nuclei, hit status, fraction of nuclei in the P1 cell population (G1 cells), in the P2 cell population (G2 cells), in the P3 cell population (hyperploid cells), as well as the ratio P1/P2 (P1 to P2), robust z-scores of these cell cycle parameters and hit status. For further explanation see Online Resource 8 (S1 Text). (XLSX 21762 kb)

Supplementary file2 S1 Figure. Distribution and reproducibility of the B-scores of cell counts obtained in the RNAi screen. Images acquired after dsRNA treatment of S2R+ cells at either the near optimal (27°C) or low temperature (17°C) were used for determination of a cell count for each well, followed by calculation of the B-score of this count. The data from negative and positive control wells (3232 of 23040 in total) are included in the graphs. (A) For each of the four replicates, B-scores were plotted for all the 60 plates, ordered according to plate number. No outlier plates were revealed. (B) For each of the four replicates, all B-scores were plotted, ordered by their magnitude. Red lines indicate the B-score limits of -2 and +2, which were used for initial hit selection. Around 20% of the outliers with B-scores below -2 are contributed by the positive control wells. (C) Reproducibility of the B-scores in the two replicates that were analyzed at the same temperature, i.e., at either 17°C (left) or 27°C (right). (PDF 4423 kb)

Supplementary file3 S2 Table. Hit lists generated based on the B-scores of the number of nuclei per well detected in the RNAi screen. The distinct hit lists (l17weak, l17weak_a, l17strong, l27strong, l_both_weak, and l_both strong) are presented on separate worksheets. For further explanation see Online Resource 8 (S1 Text). (XLSX 1011 kb)

Supplementary file4 S3 Table. Hit lists generated based on the Z-scores of cell cycle profile parameters detected in the RNAi screen. The distinct hit lists (l17caP1 to P2, l27caP1 to P2, l17caP1, l17caP2, l17caP3, l27caP1, l27caP2, and l27caP3) are presented on separate worksheets. For further explanation see Online Resource 8 (S1 Text). (XLSX 2589 kb)

Supplementary file5 S2 Figure. Depletion of control genes at 17 and 27°C results in temperature-independent effects on the cell cycle profile. Cells were treated with dsRNAs at either 17 or 27°C before DNA staining with propidium iodide (PI) and flow cytometry. In negative control experiments, either no dsRNA of *lacZ* dsRNA was added. Positive control experiments involved knockdown of well-known cell cycle regulators (*CycE*, *stg*, *Cdk1*). In case of *Cdk1*, the G2 arrest was somewhat more pronounced at 27°C, so that the minor G1 peak was no longer recognized algorithmically, precluding a calculation of the 17°C/27°C ratio displayed in Fig. 4e. (PDF 599 kb)

Supplementary file6 S3 Figure. Depletion of Pvr and Dsor1 at 17°C results in an arrest of cell cycle progression during the G2 phase. Cell cycle profiles (same as in Fig 4c) and images obtained in the genome-wide RNAi screen are displayed to reveal the effects of knockdown of *Pvr* and *Dsor1* at 17 and 27°C. For comparison, the data obtained from negative control wells (no dsRNA) and after depletion of *pbl* or *CycA* is shown as well. Knockdown of *pbl*, which is required for cytokinesis, results in binucleated cells (white arrowheads) and knockdown of *CycA *induces endo-reduplication. Scale bar = 25 µm. (PDF 2063 kb)

Supplementary file7 S4 Figure. Low temperature does not reduce Ball-EGFP levels in syncytial embryos. Extracts from *g-ball-EGFP* embryos prepared after ageing at the indicated temperatures to the syncytial blastoderm stages were analyzed by immunoblotting with anti-GFP and with anti-Lamin for control of loading. A lane with *w* embryo was included for control of anti-GFP specificity, and lanes with reduced (0.5x) and increased (1.5x) loading, respectively, for quantitative analysis of signal intensities. One of three independent but concurring replicate analyses is displayed. (PDF 140 kb)

Supplementary file8 S1 Text. Detailed description of procedures used for the genome-wide RNAi screen. (PDF 832 kb)

Supplementary file9 S4 Table. Primer sequences used for generation of dsRNA. Beyond information on the primers used for amplification of template DNA, which was transcribed in vitro for dsRNA generation, the table includes amplicon identifiers as in the GenomeRNAi database v17 (http://www.genomernai.org.) (Schmidt et al. 2013). (XLSX 13 kb)

Supplementary file10 S5 Table. Source data. Numerical data used for the preparation of the graphs shown in Figs. 4-7. (XLSX 98 kb)

## Data Availability

All data generated or analyzed during this study are included in this published article and its supplementary information files. All material generated in this study will be provided upon request.
