# Supplementary figures and images for: A genome-wide RNAi screen for genes important for proliferation of cultured Drosophila cells at low temperature identifies the Ball/VRK protein kinase
Source: Chromosoma. 2023 Feb 7;132(1):31–53. doi: 10.1007/s00412-023-00787-6 (PMC9981717; doi:10.1007/s00412-023-00787-6)

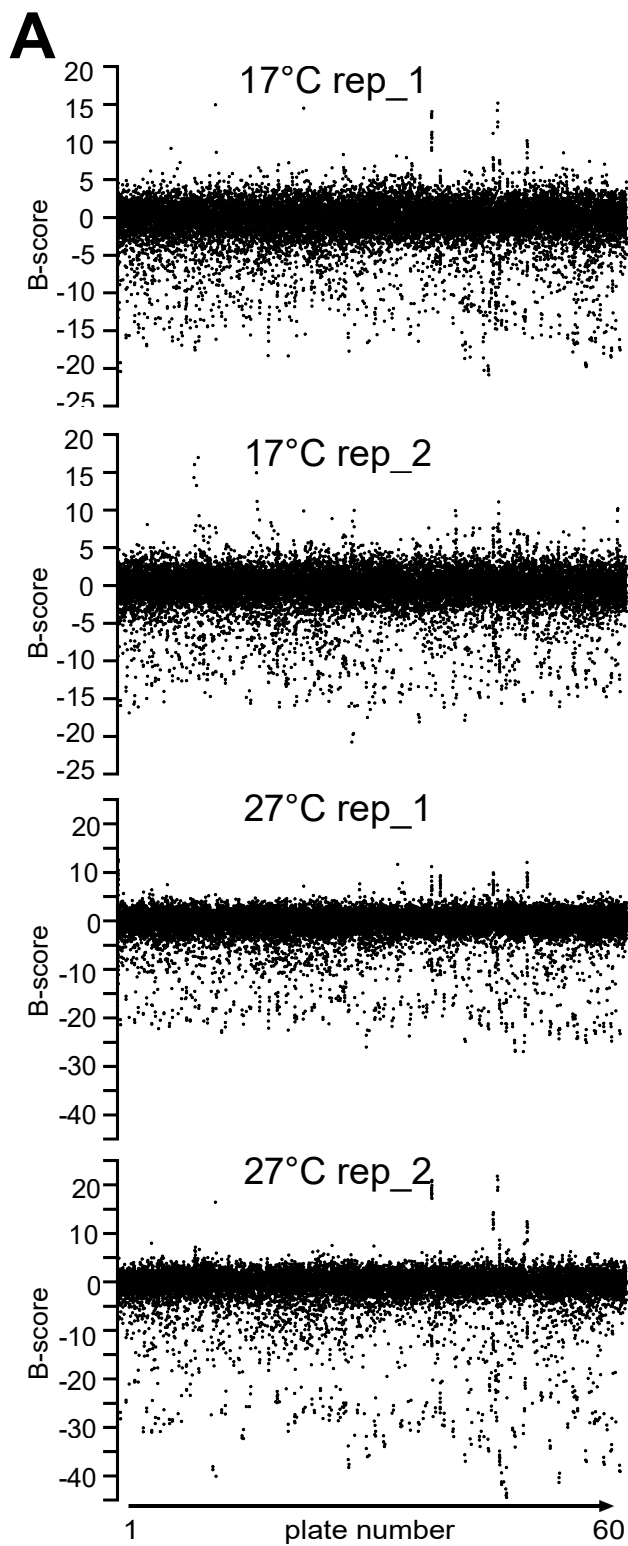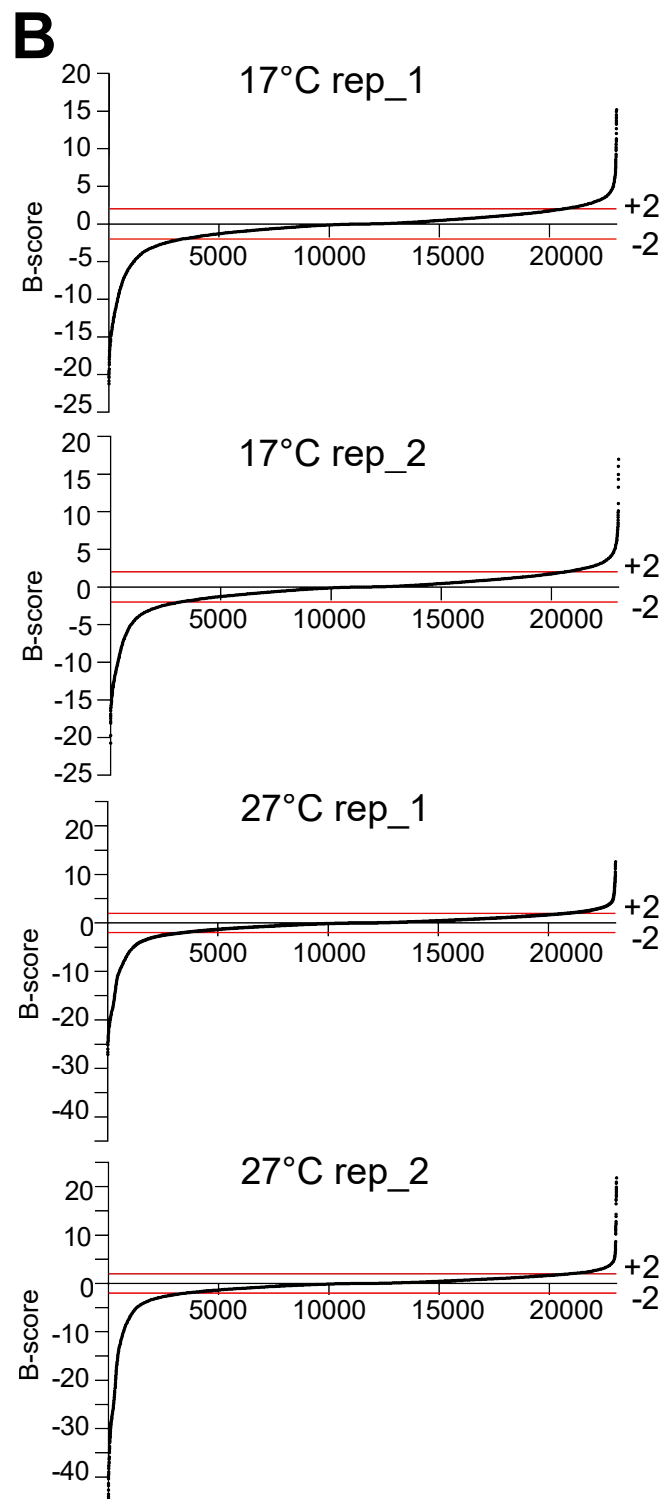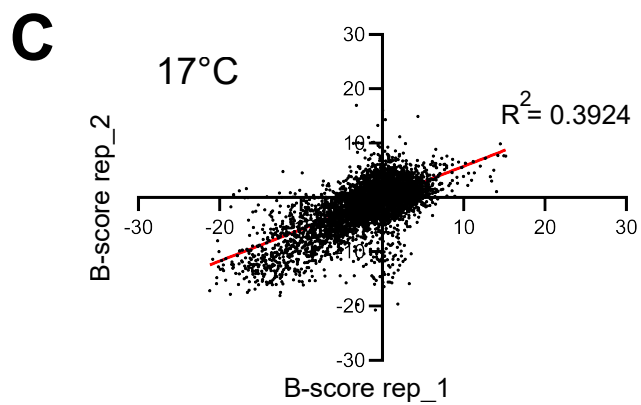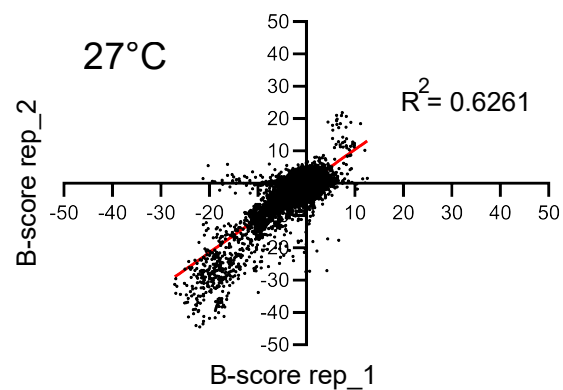

Supplement: Supplementary file 2 — Supplementary file2 S1 Figure. Distribution and reproducibility of the B-scores of cell counts obtained in the RNAi screen. Images acquired after dsRNA treatment of S2R+ cells at either the near optimal (27°C) or low temperature (17°C) were used for determination of a cell count for each well, followed by calculation of the B-score of this count. The data from negative and positive control wells (3232 of 23040 in total) are included in the graphs. (A) For each of the four replicates, B-scores were plotted for all the 60 plates, ordered according to plate number. No outlier plates were revealed. (B) For each of the four replicates, all B-scores were plotted, ordered by their magnitude. Red lines indicate the B-score limits of -2 and +2, which were used for initial hit selection. Around 20% of the outliers with B-scores below -2 are contributed by the positive control wells. (C) Reproducibility of the B-scores in the two replicates that were analyzed at the same temperature, i.e., at either 17°C (left) or 27°C (right). (PDF 4423 kb) [file 412_2023_787_MOESM2_ESM.pdf]

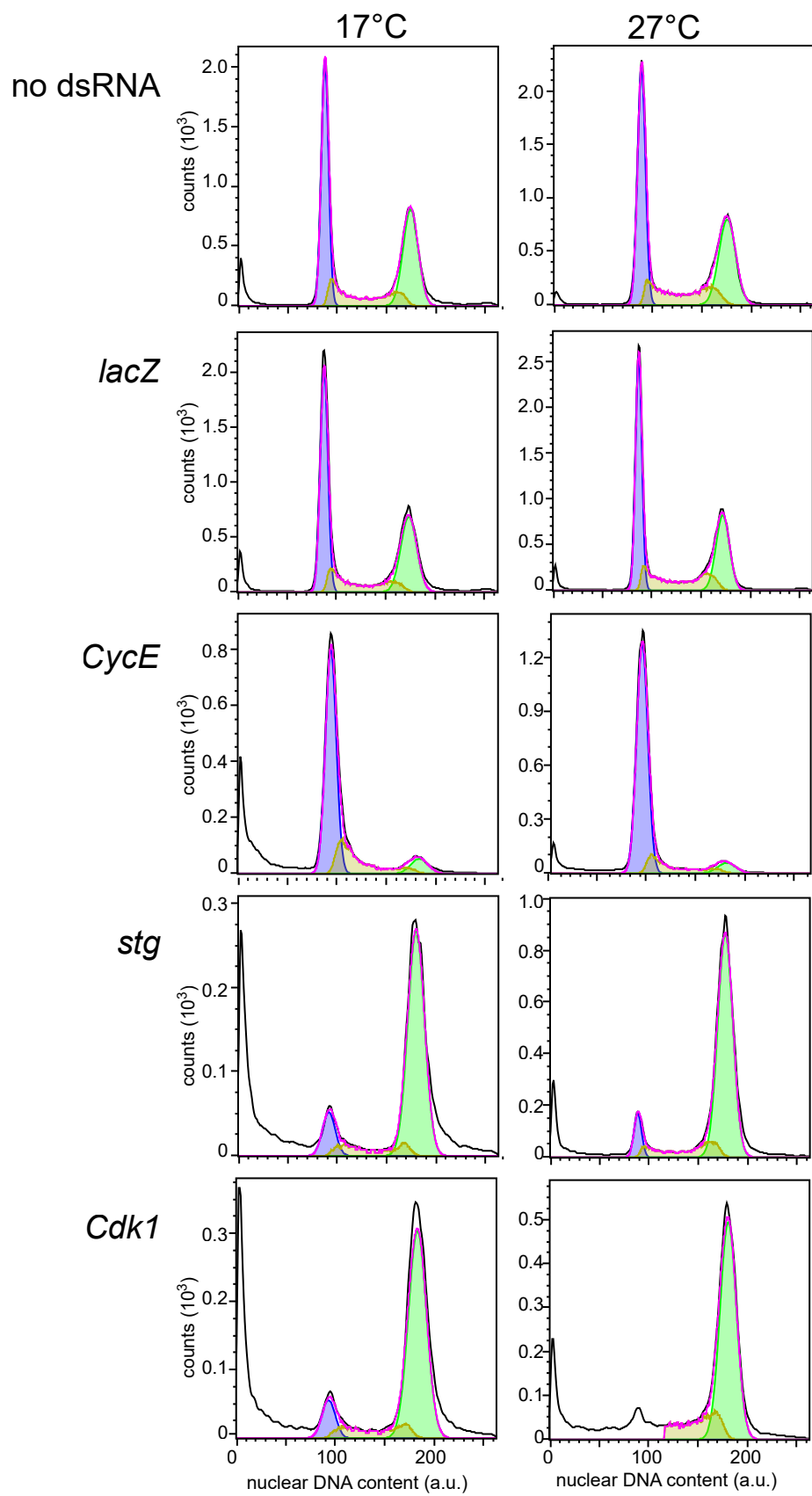

Supplement: Supplementary file 5 — Supplementary file5 S2 Figure. Depletion of control genes at 17 and 27°C results in temperature-independent effects on the cell cycle profile. Cells were treated with dsRNAs at either 17 or 27°C before DNA staining with propidium iodide (PI) and flow cytometry. In negative control experiments, either no dsRNA of lacZ dsRNA was added. Positive control experiments involved knockdown of well-known cell cycle regulators (CycE, stg, Cdk1). In case of Cdk1, the G2 arrest was somewhat more pronounced at 27°C, so that the minor G1 peak was no longer recognized algorithmically, precluding a calculation of the 17°C/27°C ratio displayed in Fig. 4e. (PDF 599 kb) [file 412_2023_787_MOESM5_ESM.pdf]

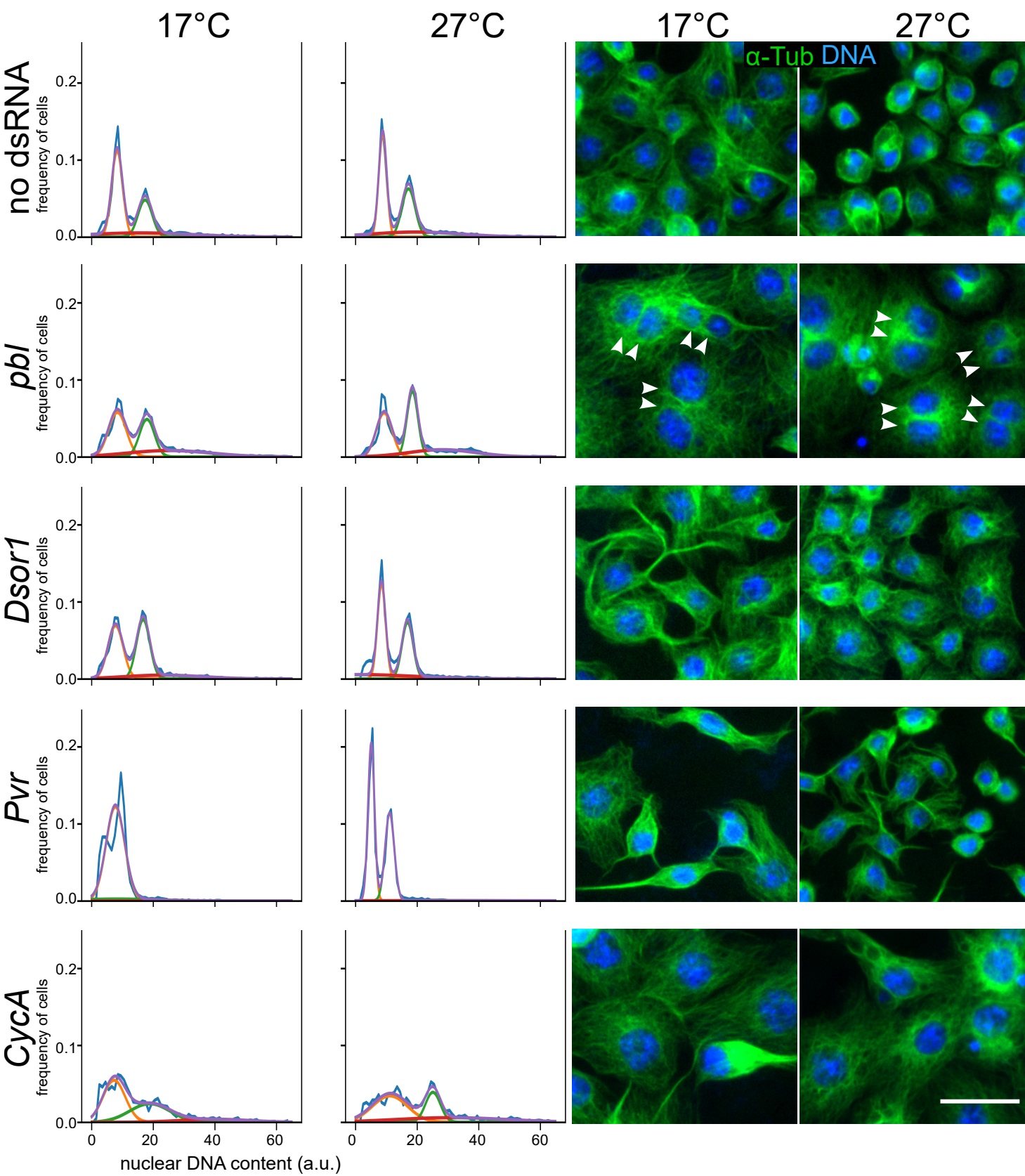

Supplement: Supplementary file 6 — Supplementary file6 S3 Figure. Depletion of Pvr and Dsor1 at 17°C results in an arrest of cell cycle progression during the G2 phase. Cell cycle profiles (same as in Fig 4c) and images obtained in the genome-wide RNAi screen are displayed to reveal the effects of knockdown of Pvr and Dsor1 at 17 and 27°C. For comparison, the data obtained from negative control wells (no dsRNA) and after depletion of pbl or CycA is shown as well. Knockdown of pbl, which is required for cytokinesis, results in binucleated cells (white arrowheads) and knockdown of CycA induces endo-reduplication. Scale bar = 25 µm. (PDF 2063 kb) [file 412_2023_787_MOESM6_ESM.pdf]

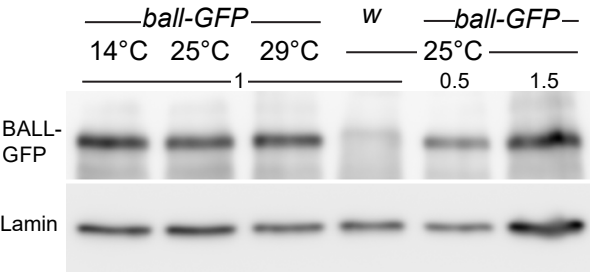

Supplement: Supplementary file 7 — Supplementary file7 S4 Figure. Low temperature does not reduce Ball-EGFP levels in syncytial embryos. Extracts from g-ball-EGFP embryos prepared after ageing at the indicated temperatures to the syncytial blastoderm stages were analyzed by immunoblotting with anti-GFP and with anti-Lamin for control of loading. A lane with w embryo was included for control of anti-GFP specificity, and lanes with reduced (0.5x) and increased (1.5x) loading, respectively, for quantitative analysis of signal intensities. One of three independent but concurring replicate analyses is displayed. (PDF 140 kb) [file 412_2023_787_MOESM7_ESM.pdf]
