# Supplementary material for: A genome-wide RNAi screen for genes important for proliferation of cultured Drosophila cells at low temperature identifies the Ball/VRK protein kinase
Source: Chromosoma. 2023 Feb 7;132(1):31–53. doi: 10.1007/s00412-023-00787-6 (PMC9981717; doi:10.1007/s00412-023-00787-6)
Supplement: Supplementary file 8 — Supplementary file8 S1 Text. Detailed description of procedures used for the genome-wide RNAi screen. (PDF 832 kb) [file 412_2023_787_MOESM8_ESM.pdf]

**A genome-wide RNAi screen for genes important for proliferation of cultured *Drosophila* cells at low temperature identifies the Ball/VRK protein kinase**

Anna Mendaluk[1], Emmanuel Caussinus[1], ...?, Michael Boutros[2,3], Christian F. Lehner[1,*]

1 Department of Molecular Life Science (DMLS), University of Zurich, Zurich, Switzerland

2 Division of Signaling and Functional Genomics, German Cancer Research Center (DKFZ), Heidelberg, Germany

3 BioQuant, Heidelberg University, Heidelberg, Germany

* christian.lehner@imls.uzh.ch

**S1 Text. Genome-wide RNAi screening procedure**

1. Treatment of S2R+ cells with dsRNA, fixation and staining

The first four steps of the genome-wide RNAi screen (generation of four replicates with dsRNA aliquots of the RNAi library, S2R+ cell seeding, fixation and staining) were performed as summarized schematically (Fig 1 of S1 Text) and described in additional details below.

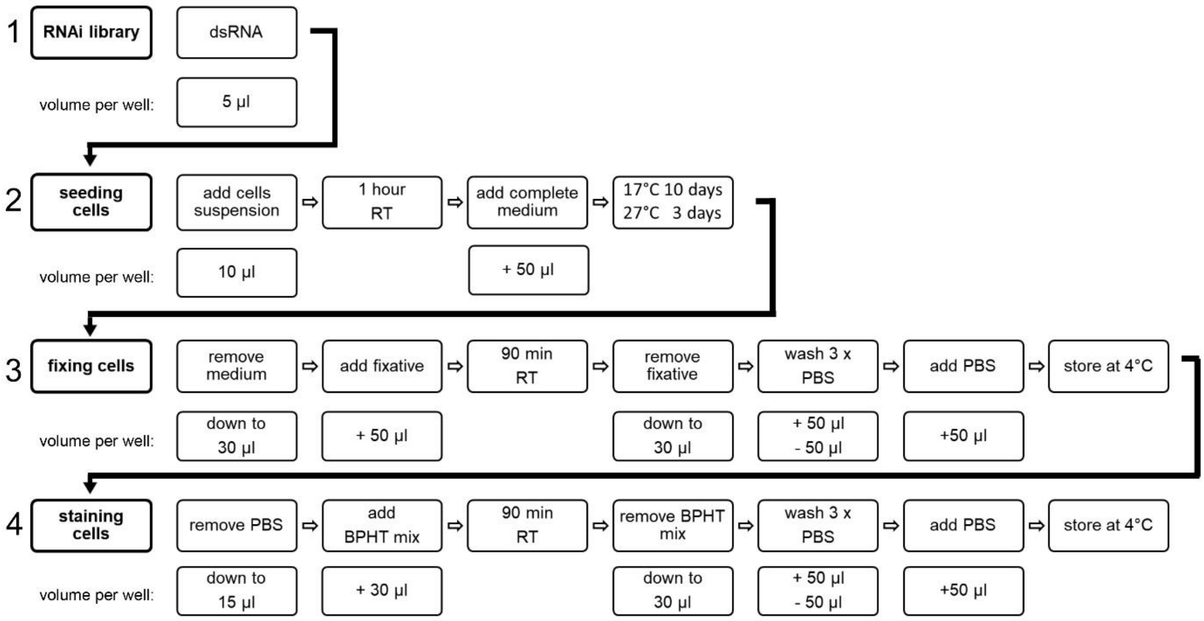


**Fig 1. S2R+ cell treatment.** Aliquots of dsRNA of the RNAi library were distributed into microplate wells to generate four replicates (step 1). S2R+ cells were added and two replicates were incubated at 17°C (for 10 days) and two replicates at 27°C (for 3 days) (step 2). After these incubations, fixation (step 3) and staining (step 4) were completed.

Step 1: Distribution of dsRNA aliquots of RNAi library. The HD2 *Drosophila* genome-wide RNAi library (Horn et al. 2010) was used. Aliquots of the dsRNA samples in this library were distributed into the wells of 384-well plates (5 µl of 50 ng/µl dsRNAs per well). Four replicates of the complete library were generated. One replicate comprised sixty 384-well plates. Every plate contained several interspersed wells that did not contain dsRNA, as well as several wells containing dsRNA targeting the anti-apoptotic gene *Diap1*.

Step 2: S2R+ cell seeding. S2R+ cells were maintained in 75-cm$^2$ cell culture flask at 25°C. Near confluent vigorously proliferating cells were harvested with Trypsin-EDTA (Gibco, #25050-014), sedimented and re-suspended in Schneider's cell culture medium (Gibco, #21720) containing 1% penicillin-streptomycin (Gibco, #15140) but no serum. After cell counting with an automated cell counter (Invitrogen, C10227), the concentration was adjusted to 1.3 x10$^6$ cells/ml. Ten µl of this cell suspension were seeded into each well using a Biomek EL406 pipetting robot, resulting in a dsRNA concentration of 16.7 µg/ml. After one hour of incubation at room temperature (RT), 50 µl of Schneider's cell culture medium containing 1% penicillin-streptomycin and 13% fetal bovine serum (FBS) (Gibco, #10500064) were added, resulting in a final concentration of 10% FBS and 200 000 cells/ml. Two replicates were incubated at 27°C for 3 days and two replicates at 17°C for 10 days in temperature-controlled incubators.

Step 3: Fixation. After incubation with dsRNA at the two distinct assay temperatures, culture medium was removed partially and formaldehyde (6.4% w/v) in phosphate buffered saline (PBS) (137 mM NaCl, 2.7 mM KCl, 1.47 mM $KH_2PO_4$, pH 7.4) was added, resulting in a final fixative concentration of 4%. Fixation was performed during 90 min at RT. Thereafter, cells were rinsed three times with PBS to remove the fixative. For storage until staining, PBS was added and the plates were kept at 4°C.

Step 4: Staining. PBS was removed and a solution (BPHT) for simultaneous blocking, permeabilization and staining with the DNA stain Hoechst 33258 and anti-Tubulin was added. BPHT consisted of PBS containing 7% FBS, 0.07% Triton X-100, 0.7 µg/ml Hoechst 33258 and fluorescein isothiocyanate (FITC)-conjugated mouse monoclonal antibody DM1A anti-α-Tubulin (Sigma, F2168) at a dilution of 1:1429. Cells were stained for 90 min at RT. Cells were then washed three times with PBS and stored in PBS at 4°C until imaging.

2. Imaging

The microplates with the stained cells were imaged at the Scientific Center for Optical and Electron Microscopy (ScopeM, ETH Zurich, Switzerland) using an automatized ImageXpress Micro High-Content Imaging System (Molecular Devices). A 4x/0.2 air objective was used for imaging of the blue channel. In addition, a 20x/0.45 air objective was used for imaging of both the blue and the green channels by acquiring single optical sections from nine partially overlapping regions from each well.

3. Hit identification based on overall DNA signal intensities

The 4x images of the DNA staining signals were used for an initial evaluation of screen data quality and initial identification of screen hits. The images were processed into whole plate images by ScopeM. In these low magnification images of entire plates, the signal intensity detected in the region of a particular well indicates, to a rough approximation, the total number of cells present in the corresponding well. Using ImageJ for image calculation, an average plate image was generated from the two replicate plates incubated at the same temperature. Visual comparison of the average plate images resulting at 17 and the 27°C allowed an identification of wells, in which cell number was low specifically at the lower temperature.

4. Determination of cell counts and cell cycle profiles by image analysis

Before image analysis of the 20x images with CellProfiler (McQuin et al. 2018), images were processed as summarized (Fig 2A of S1 Text). In a first image processing step, the effects of uneven illumination during image acquisition was corrected with ImageJ using the CIDRE correction method (Smith et al. 2015) (Fig 2B of S1 Text). The nine corrected images from the same well were then stitched into a single image with the help of ImageJ and the Plugin Grid/Collection Stitching using linear blending (Preibisch et al. 2009) (Fig 2C of S1 Text). The two fluorescence channels were stitched independently.

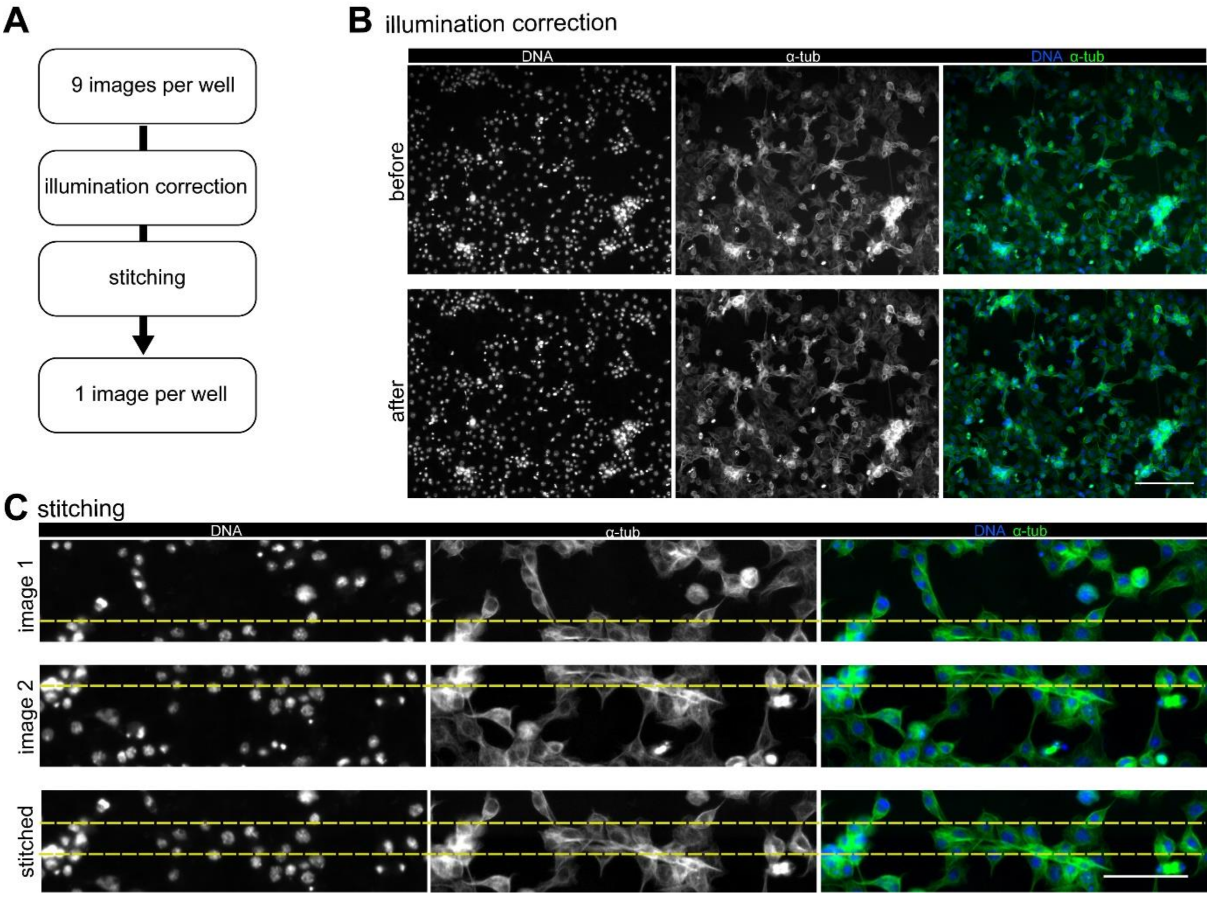


**Fig 2. Image processing.** (**A**) Overview image processing. (**B**) Step1: illumination correction. Spatially inhomogeneous illumination during microscopic image acquisition resulted in uneven signal intensities across the images, as illustrated with original raw images (top row) with a darker upper marginal region that was computationally corrected (bottom row). Scale bar = 100 µm. (**C**) Step 2: stitching. Overlap between adjacent images is marked by dashed yellow lines. The region of image 1 below the dashed yellow line is also present in image 2 above the dashed yellow line. Image overlap allowed computational stitching of the nine images from a given well into a single image, as illustrated (bottom row). Scale bar = 50 µm.

A CellProfiler pipeline (see below, 7. Cell Profiler pipeline) was used for data extraction from the stitched images. Based on DNA staining signals, cell nuclei were detected as the primary objects. Based on the anti α-Tub signals around the nuclei (primary objects), cells were detected as secondary objects. The cytoplasmic regions were the detected as tertiary objects, by deleting the nuclear regions (primary objects) from the cells (secondary objects). In addition, the integrated DNA signal intensity was determined for each detected primary object (nucleus) in a given image and the entire range of integrated DNA intensities was divided into 64 sub-ranges of even size (bins). Based on the integrated DNA intensity, each nucleus was classified into those bins. The frequency of nuclei in each bin was extracted and used for the generation DNA content histograms, i.e. cell cycle

profiles. The CellProfiler data extraction was run as a batch process using virtual machines at Service and Support for Science IT ($S^3IT$) at the University of Zurich.

5. Generation of hit lists based on cell counts

The data extracted by the CellProfiler pipeline was used for identification of screen hits. Two features were considered separately for hit identification, cell number and cell cycle profile. To create hit lists based on cell numbers (Fig 3 of S1 Text), the B-score (Brideau et al. 2003) of the cell numbers extracted for each well was calculated using a Python script (see below, 8. Python scripts). B-scores are widely used for data normalization and hit selection after in RNAi screening. The B-score indicates outlier strength (how much a particular score differs from the typical score), taking into account potential systematic variation in the measurements, like for example edge effects (i.e., variation linked to sample positions in particular rows or columns). The B-scores were calculated per plate. The B-scores obtained for the two replicates at the same temperature were averaged. Arbitrarily chosen thresholds for the average B-scores of -2 and 2 were used for the generation of distinct lists with screen hits of interest (S2 Table). The distinct hit lists were generated using the selection criteria summarized in Fig 3 of S1 Text. A first list was made with genes that appeared to be more important for cell survival/proliferation at 17°C. The dsRNA amplicons in this list, designated l17weak, resulted in a reduction of cell numbers that was unusually severe at 17°C, while at 27°C cell numbers were at most modestly reduced. A second list, l17strong, contained dsRNA amplicons that resulted in unusually high cell numbers at 17°C, while at 27°C cell numbers were at most modestly increased. Conversely, l27weak listed the dsRNA amplicons that resulted in unusually low cell counts at 27°C, while at 17°C cell numbers were at most modestly reduced. Finally, the list l27strong was made for dsRNA amplicons that resulted in unusually high cell counts at 27°C, while at 17°C cell numbers were at most modestly increased. Beyond these four lists, an additional list, l17weak_a, was generated with dsRNA amplicons resulting in unusually low cell counts at 17°C, as well as in a substantial but still lower reduction in cell numbers at 27°C (B-score ≤ -2 at both temperatures, but difference in B-scores at 17 and 27°C greater than one). Beyond these lists with hits characterized by dsRNA effects depending on temperature, two additional list were made with hits where the dsRNA had comparable effects at both analyzed temperatures. A first of these additional lists, l_both_weak contained hits characterized by unusually low cell counts at 17 and 27°C (average B-score ≤ -2 at both 17 and 27°C). The second, l_both_strong had hits of opposite character, i.e., unusually high cell counts at 17 and 27°C (average B-score ≤ 2 at both 17 and 27°C). FlyBase annotations (release 5.45) were used to assign dsRNA amplicons to genes.

The cell counts and B-scores observed for the 222 positive control wells with dsRNA targeting the anti-apoptotic *Diap1* gene provided evidence that RNAi efficiency at 17 and 27°C was comparable in our screen. In case of cell counts, the mean of the positive control wells of the four replicates was 454.0 (17_repl1), 435.5 (17_repl2), 596.9 (27_repl1) and 669.4 (27_repl2). The percentage of positive control wells with B-scores beyond the applied threshold of ≤ -2 was 98.21 (17_repl1), 96.86 (17_repl2), 96.86 (27_repl1) and 97.96 (27_repl2). The averages of the B-scores of the positive control wells were -13.23 +/- 3.73 (17_repl1), -12.16 +/- 3.32 (17_repl2), -19.00 +/- 4.67 (27_repl1) and -27.28+/- 8.00 (27_repl2).

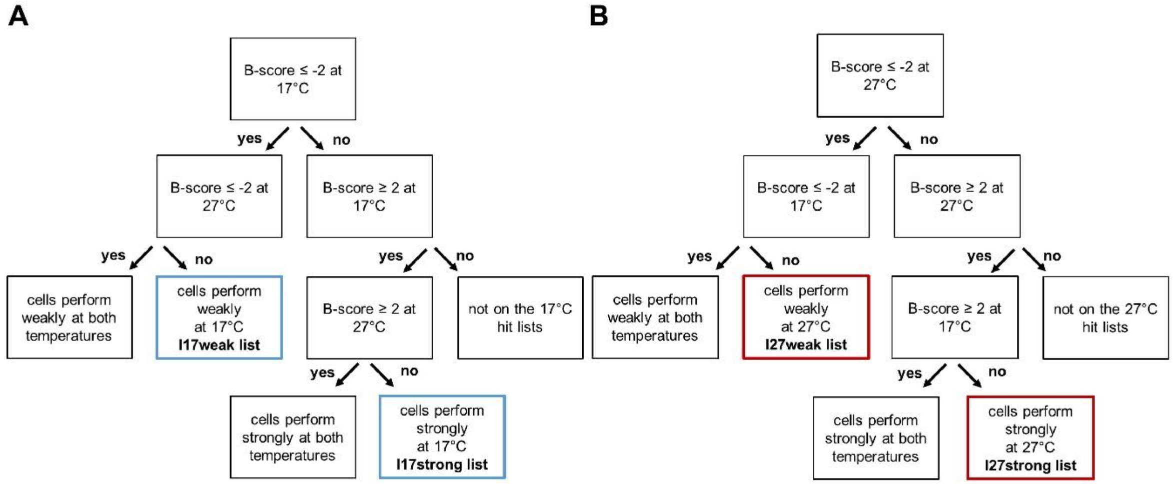


**Fig 3. Generation of lists with screen hits characterized by unusual cell counts after RNAi**

(**A,B**) Selection procedure and criteria used for the generation of hit lists based on average cell counts (mean of the two replicates for a given temperature) and their B-scores. Arbitrary chosen thresholds of -2 and 2 were used.

6. Generation of hit lists based on cell cycle profiles

For an identification of screen hits based on abnormalities of the cell cycle profile after RNAi, a histogram displaying the frequency distribution of the nuclear DNA content over the cells in a given well was generated for each well, based on the DNA staining signals. A Python script was used for creating the histograms (i.e., the cell cycle profiles), as well as for further analysis (see below, 8. Python scripts). For a parametric description of the cell cycle profile, a cell population model was fitted to the DNA signal intensity histogram profile (see also main text). Basic assumptions of this model were that the cell population in each well is comprised of three distinct Gaussian sub-populations P1, P2, and P3. In unperturbed cells, the three sub-populations correspond essentially to the G1 cells, the G2/M cells and to abnormal hyperploid cells, respectively. The frequency of cells in these three populations was calculated (P1, P2, P3) (S1 Table). Robust Z-scores were calculated for

P1, P2, P3 and for the P1/P2 ratio (S1 Table). The robust Z-scores were used for the generation of lists with screen hits (S3 Table) characterized by abnormal cell cycle profiles after RNAi. Robust Z-scores are commonly used in the analysis of high-throughput screening data (Birmingham et al. 2009). Robust z score indicates how far away (how many median absolute deviations) the value obtained for a particular sample is from the median value of all the analyzed samples. For the calculation of robust Z-scores, data of each plate were first normalized to the median value of the negative control wells included in the given plate. Thereafter, wells with highly divergent profiles between same temperature replicates were filtered out (ratio between normalized values from the two same temperature replicates <0.8 or >1.2). This filtering step was introduced because the model-fitting algorithm used for the definition of the three sub-populations P1 – P3 often failed to deliver an adequate definition of these sub-populations when abnormalities in the cell cycle profile exceeded a critical level. The reproducible data set remaining after filtering retained the values from 14093 (P1), 12654 (P2), 4298 (P3), and 7152 (P1/P2) wells out of the total 23040 wells. Robust Z-scores were then calculated for this remaining reproducible dataset, separately for each replicate, followed by averaging the same temperature replicate values. Arbitrary chosen thresholds for the average robust Z-score of -2 and +2 were used to select hits associated with an abnormal cell cycle profile primarily at one of the two tested temperatures (Fig 4 of S1 Text). The resulting lists were named l17caX (X = P1, P2, P3 or P1/P2) for dsRNA amplicons that resulted in cell cycle profile abnormalities primarily at 17°C, and conversely l27caX in case of cell cycle profile abnormalities primarily at 27°C.  The number of genes in these lists, as well as the number of associated dsRNA amplicons is presented in Table 1.

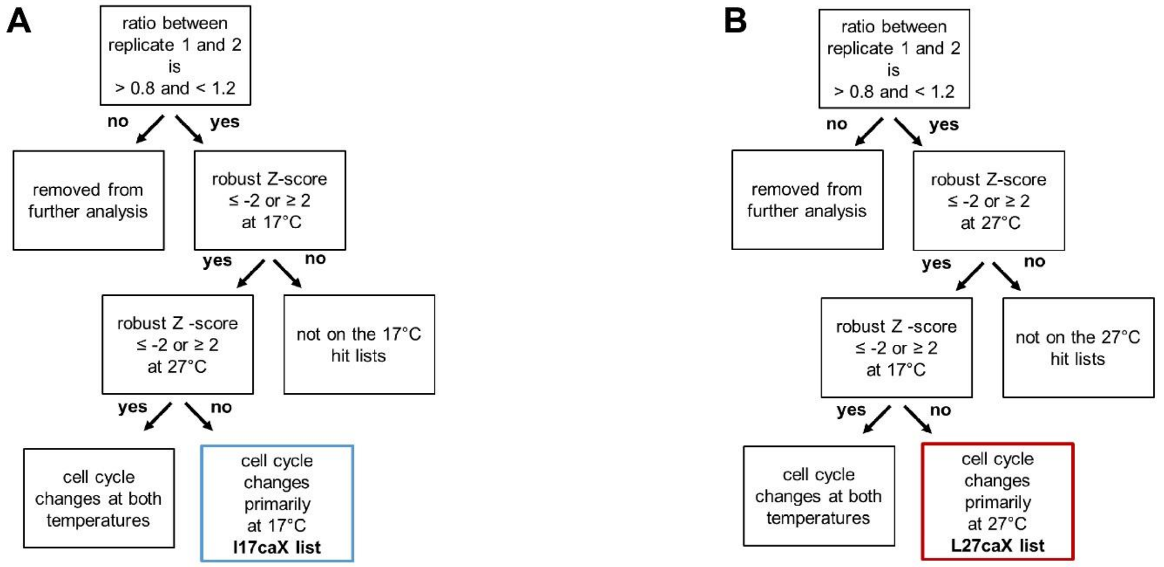


**Fig 4. Generation of lists with screen hits characterized by an abnormal cell cycle profile after RNAi**

(**A,B**) Selection procedure and criteria used for the generation of hit lists based on cell cycle profiles (DNA histograms parameters P1, P2, P3, and P1/P2 ratio).

**Table 1. Summary of hit lists based on cell cycle profile data**

| list designation | number of genes | number of dsRNA amplicons |
|---|---|---|
| l17caP1 | 1061 | 1222 |
| l17caP2 | 782 | 866 |
| l17caP3 | 425 | 459 |
| l17caP1/P2 | 700 | 774 |
| l27caP1 | 1518 | 1674 |
| l27caP2 | 1571 | 1886 |
| l27caP3 | 163 | 174 |
| l27caP1/P2 | 999 | 1125 |

For the selection of hits for validation, candidate genes on the hit lists were further evaluated. The cell cycle profiles of the 200 lowest and the 200 highest scoring genes were visually inspected for verification that the population model fit was adequate. Based on this visual inspection, six genes associated with strong cell cycle abnormalities at only one of the two temperatures were chosen for the validation experiments (*Dsor1*, *Atx2*, *nonA*, *Rack1*, *CG12090* and *Pop2*). Two additional candidate genes for validation (*Pvr* and *Pp2A-29B*) were selected with an alternative procedure introduced to overcome the limitations of the model-fitting algorithm. As already stated, the algorithm failed in defining the three sub-populations adequately when abnormalities in the cell cycle profile exceeded a critical level. In case of the original selection procedure (Fig 4 of S1 Text), an apparent very low reproducibility of the cell cycle profile parameters caused an exclusion from consideration, when only one of two same temperature replicates exceeded the critical level resulting in model fitting failure. Therefore, for selection of some additional candidate genes characterized by cell cycle profile abnormalities preferentially at one of the two assay temperatures, we used an alternative procedure without the filtering for reproducible values in the two same temperature replicates. For this alternative procedure, the values of the P1 and P2 parameters of the whole data set were normalized per plate and used for calculation of robust Z-score separately for each of two replicates tested at 17°C. The 200 highest and the 200 lowest outlier wells were identified and the corresponding top scoring dsRNA amplicons were further analyzed. For this, we evaluated the cell cycle profiles in all the wells treated with a given top scoring dsRNA amplicon, i.e., in the two tested at 17°C as well as in the two tested at 27°C. If this confirmed that abnormalities in the cell cycle

profile were obvious at 17°C but not at 27°C, the dsRNA amplicons (*Pvr*, *Pp2A-29B*) were selected for further validation.

If a cell cycle arrest occurs relatively early after addition of a particular dsRNA, the well is predicted to be associated not only with an abnormal cell cycle profile but also with a reduced cell number at the end of the treatment period. Accordingly, overlap between the genes on the hit lists based on the analyses of cell counts and of cell cycle profiles were expected and also observed. In total 167 genes identified by 192 unique dsRNA amplicons were present on both the lists with genes primarily important at 17°C for normal cell counts and normal cell cycle profiles. In case of genes primarily important at 27°C, the overlap was 274 genes identified by 316 unique dsRNA amplicons. The true extent of hit overlap in the analyses based on cell counts and cell cycle profiles is underestimated by the numbers given above, because a substantial number of values were filtered out presumably because of model fitting failures before selecting hits based on cell cycle profiles.

## 7. Cell Profiler pipelines

### 7.1. CellProfiler pipeline for RNAi screen data extraction

```
CellProfiler Pipeline: http://www.cellprofiler.org
Version:3
DateRevision:300
GitHash:
ModuleCount:23
HasImagePlaneDetails:False

Images:[module_num:1|svn_version:\'Unknown\'|variable_revision_number:2|show_window:True
|notes:\x5B\'To begin creating your project, use the Images module to compile a list of files and/or
folders that you want to analyze. You can also specify a set of rules to include only the desired files
in your selected folders.\'\x5D|batch_state:array(\x5B\x5D,
dtype=uint8)|enabled:True|wants_pause:False]
    :
    Filter images?:Custom
    Select the rule criteria:and (extension does isimage) (directory doesnot contain "Deprecated")
(directory doesnot contain "Excluded")
```

Metadata:[module_num:2|svn_version:\'Unknown\'|variable_revision_number:4|show_window:True|notes:\x5B\'The Metadata module optionally allows you to extract information describing your images (i.e, metadata) which will be stored along with your measurements. This information can be contained in the file name and/or location, or in an external file.\'\x5D|batch_state:array(\x5B\x5D, dtype=uint8)|enabled:True|wants_pause:False]

Extract metadata?:Yes

Metadata data type:Choose for each

Metadata types:{"HD2ID"\x3A "text", "FileLocation"\x3A "text", "Stitching"\x3A "text", "Well"\x3A "text", "Plate"\x3A "text", "Column"\x3A "none", "Series"\x3A "none", "Frame"\x3A "none", "MappedTargetSymbolr5.45"\x3A "text", "NR_OtherGene"\x3A "text", "Batch"\x3A "text", "Content"\x3A "text", "Reimaged"\x3A "text", "Experiment"\x3A "none", "Location"\x3A "text", "MappedTargetFBgnr5.45"\x3A "text", "MappedTargetCGr5.45"\x3A "text", "Row"\x3A "none"}

Extraction method count:5

Metadata extraction method:Extract from file/folder names

Metadata source:File name

Regular expression to extract from file name:^bHD2X0(?P<Plate>\x5B0-9\x5D{2})G(?P<Batch>\x5B0-9\x5D{2})-20x_w(?P<Well>\x5BA-P\x5D\x5B0-9\x5D{2})_s.*

Regular expression to extract from folder name:(?P<Date>\x5B0-9\x5D{4}_\x5B0-9\x5D{2}_\x5B0-9\x5D{2})$

Extract metadata from:All images

Select the filtering criteria:and (file does contain "")

Metadata file location:

Match file and image metadata:\x5B\x5D

Use case insensitive matching?:No

Metadata extraction method:Extract from file/folder names

Metadata source:Folder name

Regular expression to extract from file name:^(?P<Plate>.*)_(?P<Well>\x5BA-P\x5D\x5B0-9\x5D{2})_s(?P<Site>\x5B0-9\x5D)_w(?P<ChannelNumber>\x5B0-9\x5D)

Regular expression to extract from folder name:(?P<Reimaged>Reimaged)

Extract metadata from:All images

Select the filtering criteria:and (file does contain "")

Metadata file location:

Match file and image metadata:\x5B\x5D

Use case insensitive matching?:No

Metadata extraction method:Extract from file/folder names

Metadata source:Folder name

Regular expression to extract from file name:^(?P<Plate>.*)_(?P<Well>\x5BA-P\x5D\x5B0-9\x5D{2})_s(?P<Site>\x5B0-9\x5D)_w(?P<ChannelNumber>\x5B0-9\x5D)

Regular expression to extract from folder name:(?P<Temperature>\x5B0-9\x5D{2})_mZH

Extract metadata from:All images

Select the filtering criteria:and (file does contain "")

Metadata file location:

Match file and image metadata:\x5B\x5D

Use case insensitive matching?:No

Metadata extraction method:Import from file

Metadata source:File name

Regular expression to extract from file name:^(?P<Plate>.*)_(?P<Well>\x5BA-P\x5D\x5B0-9\x5D{2})_s(?P<Site>\x5B0-9\x5D)_w(?P<ChannelNumber>\x5B0-9\x5D)

Regular expression to extract from folder name:(?P<Date>\x5B0-9\x5D{4}_\x5B0-9\x5D{2}_\x5B0-9\x5D{2})$

Extract metadata from:All images

Select the filtering criteria:and (file does contain "")

Metadata file location:/Volumes/Stitched_1/CellProfiler_helper_files/20160713_HD2_genomewide_annotation_assayplates384.csv

Match file and image metadata:\x5B{\'Image Metadata\'\x3A u\'Plate\', \'CSV Metadata\'\x3A u\'Plate_HD2\'}, {\'Image Metadata\'\x3A u\'Well\', \'CSV Metadata\'\x3A u\'Well_HD2\'}\x5D

Use case insensitive matching?:No

Metadata extraction method:Import from file

Metadata source:File name

Regular expression to extract from file name:^(?P<Plate>.*)_(?P<Well>\x5BA-P\x5D\x5B0-9\x5D{2})_s(?P<Site>\x5B0-9\x5D)_w(?P<ChannelNumber>\x5B0-9\x5D)

Regular expression to extract from folder name:(?P<Date>\x5B0-9\x5D{4}_\x5B0-9\x5D{2}_\x5B0-9\x5D{2})$

Extract metadata from:All images

Select the filtering criteria:and (file does contain "")

Metadata file location:/Volumes/Stitched_1/CellProfiler_helper_files/Stitching-issues_all.csv

Match file and image metadata:\x5B{\'Image Metadata\'\x3A u\'Batch\', \'CSV Metadata\'\x3A u\'Batch_St\'}, {\'Image Metadata\'\x3A u\'Plate\', \'CSV Metadata\'\x3A u\'Plate_St\'}, {\'Image Metadata\'\x3A u\'Well\', \'CSV Metadata\'\x3A u\'Well_St\'}\x5D
Use case insensitive matching?:No

NamesAndTypes:[module_num:3|svn_version:\'Unknown\'|variable_revision_number:8|show_window:True|notes:\x5B\'The NamesAndTypes module allows you to assign a meaningful name to each image by which other modules will refer to it.\'\x5D|batch_state:array(\x5B\x5D, dtype=uint8)|enabled:True|wants_pause:False]
Assign a name to:Images matching rules
Select the image type:Grayscale image
Name to assign these images:DNA
Match metadata:\x5B{u\'DNA\'\x3A u\'Batch\', u\'Microtubules\'\x3A u\'Batch\'}, {u\'DNA\'\x3A u\'Plate\', u\'Microtubules\'\x3A u\'Plate\'}, {u\'DNA\'\x3A u\'Well\', u\'Microtubules\'\x3A u\'Well\'}\x5D
Image set matching method:Metadata
Set intensity range from:Image metadata
Assignments count:2
Single images count:0
Maximum intensity:16383.0
Process as 3D?:No
Relative pixel spacing in X:1.0
Relative pixel spacing in Y:1.0
Relative pixel spacing in Z:1.0
Select the rule criteria:and (file does contain "cDAPI")
Name to assign these images:DNA
Name to assign these objects:Cell
Select the image type:Grayscale image
Set intensity range from:Image bit-depth
Maximum intensity:255.0
Select the rule criteria:and (file does contain "cGFP")
Name to assign these images:Microtubules
Name to assign these objects:Cell
Select the image type:Grayscale image

Set intensity range from:Image bit-depth
Maximum intensity:255.0

Groups:[module_num:4|svn_version:\'Unknown\'|variable_revision_number:2|show_window:True|notes:\x5B\'The Groups module optionally allows you to split your list of images into image subsets (groups) which will be processed independently of each other. Examples of groupings include screening batches, microtiter plates, time-lapse movies, etc.\'\x5D|batch_state:array(\x5B\x5D, dtype=uint8)|enabled:True|wants_pause:False]
Do you want to group your images?:No
grouping metadata count:1
Metadata category:None

Crop:[module_num:5|svn_version:\'Unknown\'|variable_revision_number:3|show_window:True|notes:\x5B\x5D|batch_state:array(\x5B\x5D, dtype=uint8)|enabled:True|wants_pause:False]
Select the input image:DNA
Name the output image:CroppedDNA
Select the cropping shape:Rectangle
Select the cropping method:Coordinates
Apply which cycle\'s cropping pattern?:Every
Left and right rectangle positions:65,-65
Top and bottom rectangle positions:95,-95
Coordinates of ellipse center:500,500
Ellipse radius, X direction:400
Ellipse radius, Y direction:200
Remove empty rows and columns?:Edges
Select the masking image:None
Select the image with a cropping mask:None
Select the objects:None

Crop:[module_num:6|svn_version:\'Unknown\'|variable_revision_number:3|show_window:True|notes:\x5B\x5D|batch_state:array(\x5B\x5D, dtype=uint8)|enabled:True|wants_pause:False]
Select the input image:Microtubules
Name the output image:CroppedMicrotubules
Select the cropping shape:Previous cropping

Select the cropping method:Coordinates

Apply which cycle\'s cropping pattern?:Every

Left and right rectangle positions:65,-65

Top and bottom rectangle positions:95,-95

Coordinates of ellipse center:500,500

Ellipse radius, X direction:400

Ellipse radius, Y direction:200

Remove empty rows and columns?:Edges

Select the masking image:None

Select the image with a cropping mask:CroppedDNA

Select the objects:None

IdentifyPrimaryObjects:[module_num:7|svn_version:\'Unknown\'|variable_revision_number:13|show_window:True|notes:\x5B\x5D|batch_state:array(\x5B\x5D, dtype=uint8)|enabled:True|wants_pause:False]

Select the input image:CroppedDNA

Name the primary objects to be identified:NucleiAndMitoticFigures

Typical diameter of objects, in pixel units (Min,Max):10,45

Discard objects outside the diameter range?:Yes

Discard objects touching the border of the image?:Yes

Method to distinguish clumped objects:Intensity

Method to draw dividing lines between clumped objects:Intensity

Size of smoothing filter:10

Suppress local maxima that are closer than this minimum allowed distance:7.0

Speed up by using lower-resolution image to find local maxima?:Yes

Fill holes in identified objects?:After declumping only

Automatically calculate size of smoothing filter for declumping?:Yes

Automatically calculate minimum allowed distance between local maxima?:Yes

Handling of objects if excessive number of objects identified:Erase

Maximum number of objects:20000

Use advanced settings?:Yes

Threshold setting version:9

Threshold strategy:Global

Thresholding method:Otsu

Threshold smoothing scale:1.3488

Threshold correction factor:1.0

Lower and upper bounds on threshold:0.01,1.0

Manual threshold:0.0

Select the measurement to threshold with:None

Two-class or three-class thresholding?:Two classes

Assign pixels in the middle intensity class to the foreground or the background?:Foreground

Size of adaptive window:10

Lower outlier fraction:0.05

Upper outlier fraction:0.05

Averaging method:Mean

Variance method:Standard deviation

# of deviations:2

Thresholding method:Otsu

IdentifySecondaryObjects:[module_num:8|svn_version:\'Unknown\'|variable_revision_number:10|show_window:True|notes:\x5B\x5D|batch_state:array(\x5B\x5D, dtype=uint8)|enabled:True|wants_pause:False]

Select the input objects:NucleiAndMitoticFigures

Name the objects to be identified:CellsSpindlesFurrows

Select the method to identify the secondary objects:Propagation

Select the input image:CroppedMicrotubules

Number of pixels by which to expand the primary objects:10

Regularization factor:0.00

Discard secondary objects touching the border of the image?:No

Discard the associated primary objects?:Yes

Name the new primary objects:FilteredNucleiAndMitoticFigures

Fill holes in identified objects?:Yes

Threshold setting version:9

Threshold strategy:Global

Thresholding method:Otsu

Threshold smoothing scale:1.3488

Threshold correction factor:0.6

Lower and upper bounds on threshold:0.0,1.0

Manual threshold:0.0
Select the measurement to threshold with:None
Two-class or three-class thresholding?:Two classes
Assign pixels in the middle intensity class to the foreground or the background?:Foreground
Size of adaptive window:10
Lower outlier fraction:0.05
Upper outlier fraction:0.05
Averaging method:Mean
Variance method:Standard deviation
# of deviations:2
Thresholding method:Otsu

IdentifyTertiaryObjects:[module_num:9|svn_version:\'Unknown\'|variable_revision_number:3|show_window:True|notes:\x5B\x5D|batch_state:array(\x5B\x5D, dtype=uint8)|enabled:True|wants_pause:False]
Select the larger identified objects:CellsSpindlesFurrows
Select the smaller identified objects:NucleiAndMitoticFigures
Name the tertiary objects to be identified:Cytoplasm
Shrink smaller object prior to subtraction?:Yes

MeasureImageAreaOccupied:[module_num:10|svn_version:\'Unknown\'|variable_revision_number:4|show_window:True|notes:\x5B\x5D|batch_state:array(\x5B\x5D, dtype=uint8)|enabled:True|wants_pause:False]
Hidden:3
Measure the area occupied in a binary image, or in objects?:Objects
Select objects to measure:NucleiAndMitoticFigures
Select a binary image to measure:None
Measure the area occupied in a binary image, or in objects?:Objects
Select objects to measure:CellsSpindlesFurrows
Select a binary image to measure:None
Measure the area occupied in a binary image, or in objects?:Objects
Select objects to measure:Cytoplasm
Select a binary image to measure:None

MeasureImageIntensity:[module_num:11|svn_version:\'Unknown\'|variable_revision_number:2|sh
ow_window:True|notes:\x5B\x5D|batch_state:array(\x5B\x5D,
dtype=uint8)|enabled:True|wants_pause:False]
    Select the image to measure:CroppedDNA
    Measure the intensity only from areas enclosed by objects?:Yes
    Select the input objects:NucleiAndMitoticFigures
    Select the image to measure:CroppedMicrotubules
    Measure the intensity only from areas enclosed by objects?:Yes
    Select the input objects:Cytoplasm

MeasureObjectIntensity:[module_num:12|svn_version:\'Unknown\'|variable_revision_number:3|s
how_window:True|notes:\x5B\x5D|batch_state:array(\x5B\x5D,
dtype=uint8)|enabled:True|wants_pause:False]
    Hidden:1
    Select an image to measure:CroppedDNA
    Select objects to measure:NucleiAndMitoticFigures

MeasureObjectIntensity:[module_num:13|svn_version:\'Unknown\'|variable_revision_number:3|s
how_window:True|notes:\x5B\x5D|batch_state:array(\x5B\x5D,
dtype=uint8)|enabled:True|wants_pause:False]
    Hidden:1
    Select an image to measure:CroppedMicrotubules
    Select objects to measure:CellsSpindlesFurrows

MeasureObjectSizeShape:[module_num:14|svn_version:\'Unknown\'|variable_revision_number:1|
show_window:True|notes:\x5B\x5D|batch_state:array(\x5B\x5D,
dtype=uint8)|enabled:True|wants_pause:False]
    Select objects to measure:CellsSpindlesFurrows
    Select objects to measure:NucleiAndMitoticFigures
    Calculate the Zernike features?:No

MeasureObjectNeighbors:[module_num:15|svn_version:\'Unknown\'|variable_revision_number:2|
show_window:True|notes:\x5B\x5D|batch_state:array(\x5B\x5D,
dtype=uint8)|enabled:True|wants_pause:False]

Select objects to measure:CellsSpindlesFurrows

Select neighboring objects to measure:CellsSpindlesFurrows

Method to determine neighbors:Adjacent

Neighbor distance:5

Retain the image of objects colored by numbers of neighbors?:No

Name the output image:ObjectNeighborCount

Select colormap:Default

Retain the image of objects colored by percent of touching pixels?:No

Name the output image:PercentTouching

Select colormap:Default

CalculateMath:[module_num:16|svn_version:\'Unknown\'|variable_revision_number:2|show_window:True|notes:\x5B\x5D|batch_state:array(\x5B\x5D, dtype=uint8)|enabled:True|wants_pause:False]

Name the output measurement:FeretDiameterRatio

Operation:Divide

Select the numerator measurement type:Object

Select the numerator objects:NucleiAndMitoticFigures

Select the numerator measurement:AreaShape_MaxFeretDiameter

Multiply the above operand by:1.0

Raise the power of above operand by:1.0

Select the denominator measurement type:Object

Select the denominator objects:NucleiAndMitoticFigures

Select the denominator measurement:AreaShape_MinFeretDiameter

Multiply the above operand by:1.0

Raise the power of above operand by:1.0

Take log10 of result?:No

Multiply the result by:1.0

Raise the power of result by:1.0

Add to the result:0.0

Constrain the result to a lower bound?:No

Enter the lower bound:0.0

Constrain the result to an upper bound?:No

Enter the upper bound:1.0

CalculateMath:[module_num:17|svn_version:\'Unknown\'|variable_revision_number:2|show_window:True|notes:\x5B\x5D|batch_state:array(\x5B\x5D, dtype=uint8)|enabled:True|wants_pause:False]

Name the output measurement:IntensityRatio

Operation:Divide

Select the numerator measurement type:Object

Select the numerator objects:CellsSpindlesFurrows

Select the numerator measurement:Intensity_UpperQuartileIntensity_CroppedMicrotubules

Multiply the above operand by:1.0

Raise the power of above operand by:1.0

Select the denominator measurement type:Object

Select the denominator objects:CellsSpindlesFurrows

Select the denominator measurement:Intensity_MeanIntensityEdge_CroppedMicrotubules

Multiply the above operand by:1.0

Raise the power of above operand by:1.0

Take log10 of result?:No

Multiply the result by:1.0

Raise the power of result by:1.0

Add to the result:0.0

Constrain the result to a lower bound?:No

Enter the lower bound:0.0

Constrain the result to an upper bound?:No

Enter the upper bound:1.0

CalculateMath:[module_num:18|svn_version:\'Unknown\'|variable_revision_number:2|show_window:True|notes:\x5B\x5D|batch_state:array(\x5B\x5D, dtype=uint8)|enabled:True|wants_pause:False]

Name the output measurement:TransferedIntensityRatio

Operation:Add

Select the first operand measurement type:Object

Select the first operand objects:CellsSpindlesFurrows

Select the first operand measurement:Math_IntensityRatio

Multiply the above operand by:1.0

Raise the power of above operand by:1.0

Select the second operand measurement type:Object

Select the second operand objects:NucleiAndMitoticFigures

Select the second operand measurement:Math_FeretDiameterRatio

Multiply the above operand by:0

Raise the power of above operand by:1.0

Take log10 of result?:No

Multiply the result by:1.0

Raise the power of result by:1.0

Add to the result:0.0

Constrain the result to a lower bound?:No

Enter the lower bound:0.0

Constrain the result to an upper bound?:No

Enter the upper bound:1.0

ClassifyObjects:[module_num:19|svn_version:\'Unknown\'|variable_revision_number:2|show_window:True|notes:\x5B\x5D|batch_state:array(\x5B\x5D, dtype=uint8)|enabled:True|wants_pause:False]

Make each classification decision on how many measurements?:Single measurement

Hidden:1

Select the object to be classified:NucleiAndMitoticFigures

Select the measurement to classify by:Intensity_IntegratedIntensity_CroppedDNA

Select bin spacing:Evenly spaced bins

Number of bins:64

Lower threshold:0.0

Use a bin for objects below the threshold?:No

Upper threshold:32

Use a bin for objects above the threshold?:No

Enter the custom thresholds separating the values between bins:0,1

Give each bin a name?:Yes

Enter the bin names separated by commas:DNA01,DNA02,DNA03,DNA04,DNA05,DNA06,DNA07,DNA08,DNA09,DNA10,DNA11,DNA12,DNA13,DNA14,DNA15,DNA16,DNA17,DNA18,DNA19,DNA20,DNA21,DNA22,DNA23,DNA24,DNA25,DNA26,DNA27,DNA28,DNA29,DNA30,DNA31,DNA32,DNA33,DNA34,DNA35,DNA36,DNA37,DNA38,DN

A39,DNA40,DNA41,DNA42,DNA43,DNA44,DNA45,DNA46,DNA47,DNA48,DNA49,DNA50,DNA51,DNA52,DNA53,DNA54,DNA55,DNA56,DNA57,DNA58,DNA59,DNA60,DNA61,DNA62,DNA63,DNA64

Retain an image of the classified objects?:No

Name the output image:ClassifiedNuclei

Select the object name:None

Select the first measurement:None

Method to select the cutoff:Mean

Enter the cutoff value:0.5

Select the second measurement:None

Method to select the cutoff:Mean

Enter the cutoff value:0.5

Use custom names for the bins?:No

Enter the low-low bin name:low_low

Enter the low-high bin name:low_high

Enter the high-low bin name:high_low

Enter the high-high bin name:high_high

Retain an image of the classified objects?:No

Enter the image name:None

ClassifyObjects:[module_num:20|svn_version:\'Unknown\'|variable_revision_number:2|show_window:True|notes:\x5B\x5D|batch_state:array(\x5B\x5D,
dtype=uint8)|enabled:True|wants_pause:True]

Make each classification decision on how many measurements?:Pair of measurements

Hidden:1

Select the object to be classified:None

Select the measurement to classify by:None

Select bin spacing:Evenly spaced bins

Number of bins:3

Lower threshold:0.0

Use a bin for objects below the threshold?:No

Upper threshold:1.0

Use a bin for objects above the threshold?:No

Enter the custom thresholds separating the values between bins:0,1

Give each bin a name?:No

Enter the bin names separated by commas:None
Retain an image of the classified objects?:No
Name the output image:ClassifiedNuclei
Select the object name:NucleiAndMitoticFigures
Select the first measurement:Math_FeretDiameterRatio
Method to select the cutoff:Custom
Enter the cutoff value:1.5
Select the second measurement:Math_TransferedIntensityRatio
Method to select the cutoff:Custom
Enter the cutoff value:3
Use custom names for the bins?:Yes
Enter the low-low bin name:Interphasic
Enter the low-high bin name:LowFRatio
Enter the high-low bin name:LowIRatio
Enter the high-high bin name:Metaphasic
Retain an image of the classified objects?:Yes
Enter the image name:MetaphasicFigures

ExportToSpreadsheet:[module_num:21|svn_version:\'Unknown\'|variable_revision_number:12|show_window:True|notes:\x5B\x5D|batch_state:array(\x5B\x5D, dtype=uint8)|enabled:True|wants_pause:False]
Select the column delimiter:Comma (",")
Add image metadata columns to your object data file?:No
Select the measurements to export:Yes
Calculate the per-image mean values for object measurements?:No
Calculate the per-image median values for object measurements?:No
Calculate the per-image standard deviation values for object measurements?:No
Output file location:Default Output Folder\x7C
Create a GenePattern GCT file?:No
Select source of sample row name:Metadata
Select the image to use as the identifier:None
Select the metadata to use as the identifier:None
Export all measurement types?:No

Press button to select measurements:Image\x7CClassify_DNA20_NumObjectsPerBin,Image\x7CClassify_DNA21_NumObjectsPerBin,Image\x7CClassify_DNA22_NumObjectsPerBin,Image\x7CClassify_DNA23_NumObjectsPerBin,Image\x7CClassify_DNA24_NumObjectsPerBin,Image\x7CClassify_DNA25_NumObjectsPerBin,Image\x7CClassify_DNA26_NumObjectsPerBin,Image\x7CClassify_DNA27_NumObjectsPerBin,Image\x7CClassify_DNA28_NumObjectsPerBin,Image\x7CClassify_DNA29_NumObjectsPerBin,Image\x7CClassify_DNA49_NumObjectsPerBin,Image\x7CClassify_DNA07_NumObjectsPerBin,Image\x7CClassify_DNA46_NumObjectsPerBin,Image\x7CClassify_DNA64_NumObjectsPerBin,Image\x7CClassify_DNA08_NumObjectsPerBin,Image\x7CClassify_DNA45_NumObjectsPerBin,Image\x7CClassify_DNA42_NumObjectsPerBin,Image\x7CClassify_DNA43_NumObjectsPerBin,Image\x7CClassify_DNA40_NumObjectsPerBin,Image\x7CClassify_DNA41_NumObjectsPerBin,Image\x7CClassify_DNA02_NumObjectsPerBin,Image\x7CClassify_DNA03_NumObjectsPerBin,Image\x7CClassify_DNA01_NumObjectsPerBin,Image\x7CClassify_DNA60_NumObjectsPerBin,Image\x7CClassify_DNA61_NumObjectsPerBin,Image\x7CClassify_DNA48_NumObjectsPerBin,Image\x7CClassify_DNA63_NumObjectsPerBin,Image\x7CClassify_DNA15_NumObjectsPerBin,Image\x7CClassify_DNA04_NumObjectsPerBin,Image\x7CClassify_DNA58_NumObjectsPerBin,Image\x7CClassify_DNA62_NumObjectsPerBin,Image\x7CClassify_DNA05_NumObjectsPerBin,Image\x7CClassify_DNA47_NumObjectsPerBin,Image\x7CClassify_DNA37_NumObjectsPerBin,Image\x7CClassify_DNA36_NumObjectsPerBin,Image\x7CClassify_DNA35_NumObjectsPerBin,Image\x7CClassify_DNA34_NumObjectsPerBin,Image\x7CClassify_DNA33_NumObjectsPerBin,Image\x7CClassify_DNA32_NumObjectsPerBin,Image\x7CClassify_DNA31_NumObjectsPerBin,Image\x7CClassify_DNA30_NumObjectsPerBin,Image\x7CClassify_DNA39_NumObjectsPerBin,Image\x7CClassify_DNA38_NumObjectsPerBin,Image\x7CClassify_DNA55_NumObjectsPerBin,Image\x7CClassify_DNA18_NumObjectsPerBin,Image\x7CClassify_DNA57_NumObjectsPerBin,Image\x7CClassify_DNA56_NumObjectsPerBin,Image\x7CClassify_DNA51_NumObjectsPerBin,Image\x7CClassify_DNA50_NumObjectsPerBin,Image\x7CClassify_DNA53_NumObjectsPerBin,Image\x7CClassify_DNA52_NumObjectsPerBin,Image\x7CClassify_DNA11_NumObjectsPerBin,Image\x7CClassify_DNA10_NumObjectsPerBin,Image\x7CClassify_DNA13_NumObjectsPerBin,Image\x7CClassify_DNA12_NumObjectsPerBin,Image\x7CClassify_DNA59_NumObjectsPerBin,Image\x7CClassify_DNA14_NumObjectsPerBin,Image\x7CClassify_DNA17_NumObjectsPerBin,Image\x7CClassify_DNA16_NumObjectsPerBin,Image\x7CClassify_DNA19_NumObjectsPerBin,Image\x7CClassify_DNA54_NumObjectsPerBin,Image\x7CClassify_DNA44_NumObjectsPerBin,Image\x7CClassify_DNA06_NumObjectsPerBin,Image\x7CClassify_DNA09_NumObjectsPerBin

Representation of Nan/Inf:NaN

Add a prefix to file names?:Yes

Filename prefix:DNA_Histogram_

Overwrite existing files without warning?:Yes

Data to export:Image

Combine these object measurements with those of the previous object?:No

File name:\\\\g<Temperature>\\u00b0C_Batch_\\\\g<Batch>_Plate_\\\\g<Plate>_Well_\\\\g<Well>.csv

Use the object name for the file name?:No

ExportToDatabase:[module_num:22|svn_version:\'Unknown\'|variable_revision_number:27|show_window:True|notes:\x5B\x5D|batch_state:array(\x5B\x5D, dtype=uint8)|enabled:True|wants_pause:False]

Database type:MySQL / CSV

Database name:RNAiScreen

Add a prefix to table names?:No

Table prefix:MyExpt_

SQL file prefix:SQL_

Output file location:Default Output Folder\x7C

Create a CellProfiler Analyst properties file?:No

Database host:

Username:

Password:

Name the SQLite database file:DefaultDB.db

Calculate the per-image mean values of object measurements?:No

Calculate the per-image median values of object measurements?:No

Calculate the per-image standard deviation values of object measurements?:No

Calculate the per-well mean values of object measurements?:Yes

Calculate the per-well median values of object measurements?:Yes

Calculate the per-well standard deviation values of object measurements?:Yes

Export measurements for all objects to the database?:None

Select the objects:CellsSpindlesFurrows

Maximum # of characters in a column name:64

Create one table per object, a single object table or a single object view?:Single object table

Enter an image url prepend if you plan to access your files via http:

Write image thumbnails directly to the database?:No

Select the images for which you want to save thumbnails:

Auto-scale thumbnail pixel intensities?:Yes

Select the plate type:384

Select the plate metadata:Plate

Select the well metadata:Well

Include information for all images, using default values?:No

Properties image group count:1

Properties group field count:1

Properties filter field count:0

Workspace measurement count:1

Experiment name:RNAiScreenPipeV1

Which objects should be used for locations?:Cytoplasm

Enter a phenotype class table name if using the Classifier tool in CellProfiler Analyst:

Export object relationships?:No

Overwrite without warning?:Never

Access CellProfiler Analyst images via URL?:No

Select the classification type:Object

Select an image to include:Microtubules

Use the image name for the display?:Yes

Image name:Channel1

Channel color:red

Do you want to add group fields?:No

Enter the name of the group:

Enter the per-image columns which define the group, separated by commas:ImageNumber, Image_Metadata_Plate, Image_Metadata_Well

Do you want to add filter fields?:No

Automatically create a filter for each plate?:No

Create a CellProfiler Analyst workspace file?:No

Select the measurement display tool:ScatterPlot

Type of measurement to plot on the X-axis:Image

Enter the object name:None

Select the X-axis measurement:None

Select the X-axis index:ImageNumber

Type of measurement to plot on the Y-axis:Image

Enter the object name:None
Select the Y-axis measurement:None
Select the Y-axis index:ImageNumber

CreateBatchFiles:[module_num:23|svn_version:\'Unknown\'|variable_revision_number:8|show_window:True|notes:\x5B\x5D|batch_state:array(\x5B\x5D, dtype=uint8)|enabled:True|wants_pause:False]
Store batch files in default output folder?:Yes
Output folder path:/Users/emmanuelc/CellProfiler_sandbox
Are the cluster computers running Windows?:No
Hidden\x3A in batch mode:No
Hidden\x3A in distributed mode:No
Hidden\x3A default input folder at time of save:/Users/emmanuelc/CellProfiler_sandbox
Hidden\x3A revision number:0
Hidden\x3A from old matlab:No
Local root path:/Volumes/Stitched_1
Cluster root path:/disk_input

7.2. CellProfiler pipeline for data extraction in validation experiments

CellProfiler Pipeline: http://www.cellprofiler.org
Version:3
DateRevision:20160503183100
GitHash:ac0529e
ModuleCount:9
HasImagePlaneDetails:False

Images:[module_num:1|svn_version:\'Unknown\'|variable_revision_number:2|show_window:False|notes:\x5B\'To begin creating your project, use the Images module to compile a list of files and/or folders that you want to analyze. You can also specify a set of rules to include only the desired files in your selected folders.\'\x5D|batch_state:array(\x5B\x5D, dtype=uint8)|enabled:True|wants_pause:False]
:
Filter images?:Images only

Select the rule criteria:and (extension does isimage) (directory doesnot containregexp "\x5B\\\\\\\\\\\\\\\\/\x5D\\\\\\\\.")

Metadata:[module_num:2|svn_version:\'Unknown\'|variable_revision_number:4|show_window:False|notes:\x5B\'The Metadata module optionally allows you to extract information describing your images (i.e, metadata) which will be stored along with your measurements. This information can be contained in the file name and/or location, or in an external file.\'\x5D|batch_state:array(\x5B\x5D, dtype=uint8)|enabled:True|wants_pause:False]

Extract metadata?:No

Metadata data type:Text

Metadata types:{}

Extraction method count:1

Metadata extraction method:Extract from file/folder names

Metadata source:File name

Regular expression:^(?P<Plate>.*)_(?P<Well>\x5BA-P\x5D\x5B0-9\x5D{2})_s(?P<Site>\x5B0-9\x5D)_w(?P<ChannelNumber>\x5B0-9\x5D)

Regular expression:(?P<Date>\x5B0-9\x5D{4}_\x5B0-9\x5D{2}_\x5B0-9\x5D{2})$

Extract metadata from:All images

Select the filtering criteria:and (file does contain "")

Metadata file location:

Match file and image metadata:\x5B\x5D

Use case insensitive matching?:No

NamesAndTypes:[module_num:3|svn_version:\'Unknown\'|variable_revision_number:6|show_window:False|notes:\x5B\'The NamesAndTypes module allows you to assign a meaningful name to each image by which other modules will refer to it.\'\x5D|batch_state:array(\x5B\x5D, dtype=uint8)|enabled:True|wants_pause:False]

Assign a name to:Images matching rules

Select the image type:Grayscale image

Name to assign these images:DNA

Match metadata:\x5B\x5D

Image set matching method:Order

Set intensity range from:Image metadata

Assignments count:2

Single images count:0
Maximum intensity:255.0
Select the rule criteria:and (file does contain "w1") (file doesnot contain "thumb")
Name to assign these images:DNA
Name to assign these objects:Cell
Select the image type:Grayscale image
Set intensity range from:Image metadata
Retain outlines of loaded objects?:No
Name the outline image:LoadedOutlines
Maximum intensity:255.0
Select the rule criteria:and (file does contain "w2") (file doesnot contain "thumb")
Name to assign these images:GFP
Name to assign these objects:Nucleus
Select the image type:Grayscale image
Set intensity range from:Image metadata
Retain outlines of loaded objects?:No
Name the outline image:LoadedOutlines
Maximum intensity:255.0

Groups:[module_num:4|svn_version:\'Unknown\'|variable_revision_number:2|show_window:False|notes:\x5B\'The Groups module optionally allows you to split your list of images into image subsets (groups) which will be processed independently of each other. Examples of groupings include screening batches, microtiter plates, time-lapse movies, etc.\'\x5D|batch_state:array(\x5B\x5D, dtype=uint8)|enabled:True|wants_pause:False]
Do you want to group your images?:No
grouping metadata count:1
Metadata category:None

IdentifyPrimaryObjects:[module_num:5|svn_version:\'Unknown\'|variable_revision_number:10|show_window:True|notes:\x5B\x5D|batch_state:array(\x5B\x5D, dtype=uint8)|enabled:True|wants_pause:False]
Select the input image:DNA
Name the primary objects to be identified:Nuclei
Typical diameter of objects, in pixel units (Min,Max):10,70

Discard objects outside the diameter range?:Yes

Try to merge too small objects with nearby larger objects?:No

Discard objects touching the border of the image?:Yes

Method to distinguish clumped objects:Laplacian of Gaussian

Method to draw dividing lines between clumped objects:Intensity

Size of smoothing filter:10

Suppress local maxima that are closer than this minimum allowed distance:7.0

Speed up by using lower-resolution image to find local maxima?:Yes

Name the outline image:PrimaryOutlines

Fill holes in identified objects?:After both thresholding and declumping

Automatically calculate size of smoothing filter for declumping?:Yes

Automatically calculate minimum allowed distance between local maxima?:Yes

Retain outlines of the identified objects?:No

Automatically calculate the threshold using the Otsu method?:Yes

Enter Laplacian of Gaussian threshold:0.5

Automatically calculate the size of objects for the Laplacian of Gaussian filter?:Yes

Enter LoG filter diameter:5.0

Handling of objects if excessive number of objects identified:Continue

Maximum number of objects:500

Threshold setting version:2

Threshold strategy:Global

Thresholding method:Otsu

Select the smoothing method for thresholding:Automatic

Threshold smoothing scale:1.0

Threshold correction factor:1.0

Lower and upper bounds on threshold:0.005,1.0

Approximate fraction of image covered by objects?:0.01

Manual threshold:0.5

Select the measurement to threshold with:None

Select binary image:None

Masking objects:None

Two-class or three-class thresholding?:Two classes

Minimize the weighted variance or the entropy?:Weighted variance

Assign pixels in the middle intensity class to the foreground or the background?:Foreground

Method to calculate adaptive window size:Image size
Size of adaptive window:10
Use default parameters?:Default
Lower outlier fraction:0.05
Upper outlier fraction:0.05
Averaging method:Mean
Variance method:Standard deviation
# of deviations:2.0

IdentifySecondaryObjects:[module_num:6|svn_version:\'Unknown\'|variable_revision_number:9|show_window:True|notes:\x5B\x5D|batch_state:array(\x5B\x5D, dtype=uint8)|enabled:True|wants_pause:False]
Select the input objects:Nuclei
Name the objects to be identified:Cells
Select the method to identify the secondary objects:Watershed - Image
Select the input image:GFP
Number of pixels by which to expand the primary objects:10
Regularization factor:0.05
Name the outline image:SecondaryOutlines
Retain outlines of the identified secondary objects?:No
Discard secondary objects touching the border of the image?:No
Discard the associated primary objects?:No
Name the new primary objects:FilteredNuclei
Retain outlines of the new primary objects?:No
Name the new primary object outlines:FilteredNucleiOutlines
Fill holes in identified objects?:Yes
Threshold setting version:2
Threshold strategy:Global
Thresholding method:Otsu
Select the smoothing method for thresholding:No smoothing
Threshold smoothing scale:1.0
Threshold correction factor:1.0
Lower and upper bounds on threshold:0.0,1.0
Approximate fraction of image covered by objects?:0.01

Manual threshold:0.8
Select the measurement to threshold with:None
Select binary image:None
Masking objects:None
Two-class or three-class thresholding?:Two classes
Minimize the weighted variance or the entropy?:Weighted variance
Assign pixels in the middle intensity class to the foreground or the background?:Foreground
Method to calculate adaptive window size:Image size
Size of adaptive window:10
Use default parameters?:Default
Lower outlier fraction:0.05
Upper outlier fraction:0.05
Averaging method:Mean
Variance method:Standard deviation
# of deviations:2.0

MeasureObjectIntensity:[module_num:7|svn_version:\'Unknown\'|variable_revision_number:3|show_window:True|notes:\x5B\x5D|batch_state:array(\x5B\x5D, dtype=uint8)|enabled:True|wants_pause:False]
Hidden:1
Select an image to measure:GFP
Select objects to measure:Cells

FilterObjects:[module_num:8|svn_version:\'Unknown\'|variable_revision_number:7|show_window:True|notes:\x5B\x5D|batch_state:array(\x5B\x5D, dtype=uint8)|enabled:True|wants_pause:False]
Name the output objects:FilteredGreen
Select the object to filter:Cells
Select the filtering mode:Measurements
Select the filtering method:Limits
Select the objects that contain the filtered objects:None
Retain outlines of the identified objects?:No
Name the outline image:FilteredObjects
Rules file location:Elsewhere...\x7C
Rules file name:rules.txt

Class number:1
Measurement count:1
Additional object count:0
Assign overlapping child to:Both parents
Select the measurement to filter by:Intensity_MinIntensity_GFP
Filter using a minimum measurement value?:Yes
Minimum value:0.03
Filter using a maximum measurement value?:No
Maximum value:1.0

ExportToSpreadsheet:[module_num:9|svn_version:\'Unknown\'|variable_revision_number:11|show_window:True|notes:\x5B\x5D|batch_state:array(\x5B\x5D, dtype=uint8)|enabled:True|wants_pause:False]
Select the column delimiter:Comma (",")
Add image metadata columns to your object data file?:No
Limit output to a size that is allowed in Excel?:No
Select the measurements to export:No
Calculate the per-image mean values for object measurements?:No
Calculate the per-image median values for object measurements?:No
Calculate the per-image standard deviation values for object measurements?:No
Output file location:Default Input Folder sub-folder\x7CDesktop\\\\\\\\cell profiler testing
Create a GenePattern GCT file?:No
Select source of sample row name:Metadata
Select the image to use as the identifier:None
Select the metadata to use as the identifier:None
Export all measurement types?:Yes
:
Representation of Nan/Inf:NaN
Add a prefix to file names?:Yes
Filename prefix:27_2_3
Overwrite existing files without warning?:No
Data to export:Do not use
Combine these object measurements with those of the previous object?:No
File name:DATA.csv

Use the object name for the file name?:Yes

## 8. Python scripts

### 8.1. B-score calculations

```
import pandas as pd
import numpy as np

#Given a 2D array, where [ij] is the measured signal value at row i and column j
#The B-score is calculated as follows:
#
#B-score[ij]=median_polish_cell_residue[ij]/MAD
def myMAD(dataM):
    med = np.median(dataM)
    dataM = abs(dataM-med)
    MAD = np.median(dataM)
    return MAD

#Performs median polish on a 2-D array
#returns only the cell residues
#
#in code below
#   grand_effect: µ
#   margins[0]: column effects
#   margins[1]: row effects
def median_polish(dataP: np.ndarray, n_iter: int = 10):
    assert dataP.ndim == 2, "Input must be 2D array"
    ndim = 2
    dataP = dataP.copy()
    grand_effect = np.median(dataP)
    dataP -= grand_effect
    median_margins = [0] * ndim
    margins = [np.zeros(shape=dataP.shape[idx]) for idx in range(2)]
    dim_mask = np.ones(ndim, dtype=np.int)
```

```
    for _ in range(n_iter):
        for dim_id in range(ndim):
            rest_dim = 1 - dim_id
            temp_median = np.median(dataP, rest_dim)
            margins[dim_id] += temp_median
            median_margins[rest_dim] = np.median(margins[rest_dim])
            margins[rest_dim] -= median_margins[rest_dim]
            dim_mask[dim_id] = -1
            dataP -= temp_median.reshape(dim_mask)
            dim_mask[dim_id] = 1
        grand_effect += sum(median_margins)
    return dataP

#import and read the csv files
data=pd.read_csv('G:\\rep_1_17.csv', sep=',')

plate_numbers=[1,2,3,4,5,6,7,8,9,10,11,12,13,14,15,16,17,18,19,20,21,22,23,24,25,26,27,28,29,30,3
1,32,33,34,35,36,37,38,39,40,41,42,43,44,45,46,47,48,49,50,51,52,53,54,55,56,57,58,59,60]
measurement="Image_Count_FilteredNucleiAndMitoticFigures"
column_name = measurement + "_B_score"

for i in plate_numbers:
    #choose plate by its number
    plate=data[(data["Image_Metadata_Plate"]== i)]
    plate_wells=plate[measurement].values.reshape(16,24)
    plate_wells=plate_wells.astype('float64')
    #B-score[ij]=median_polish_cell_residue[ij]/MAD
    plate_residues=median_polish(plate_wells)
    plate_residues=pd.Series(plate_residues.reshape(384))
    B_score=plate_residues/myMAD(plate_residues)
    #add normaliased data to table
    data.loc[data["Image_Metadata_Plate"]== i,column_name]=B_score.values
```

```
    print("plate:",i," is done!")

data.to_csv('G:\\b-score_17_rep_1', sep=',')
```

8.2. Calculating and extracting histogram parameters (P1, P2, P3, P1/P2)

```
import math
import numpy
import pandas
from scipy.optimize import minimize
from scipy.stats import norm
from scipy.stats import chisquare

# two lines below without '.csv'
csv_file_to_process = "/Users/rep_1_17"
extension_for_processed_file = "_result"



def fthn(m, s):
    fth = numpy.empty(64)
    # fth<-function(x,y) pnorm((c(1:64)-x)/y)-pnorm((c(0:63)-x)/y)
    for i in range(64):
        fth[i] = norm.cdf((i + 1 - m) / s) - norm.cdf((i - m) / s)

    # fthn<-function(x,y) fth(x,y)/sum(fth(x,y))
    return fth / fth.sum()



def process_one_well(row):
    yValues = [row["Image_Classify_DNA{:02d}_NumObjectsPerBin".format(i)] for i in range(1, 65)]
    yValues = numpy.array(yValues)
    print("yValues",yValues)
    n = yValues.sum()
    if n==0:
```

```
        print('temp=' + str(row['Image_Metadata_Temperature']) + ' batch=' + str(
            row['Image_Metadata_Batch']) + ' plate=' + str(row['Image_Metadata_Plate']) + ' well=' +
row[
                'Image_Metadata_Well'] + ' could not be processed')

        return 0, 0, 0, 0, 0, 0, 0, 0, 0, 0, 0
    yFreq = yValues / n
    print("yFreq",yFreq)

    average = math.floor((yFreq[:40] * numpy.arange(1, 41)).sum())
    if average == 0:
        print('temp=' + str(row['Image_Metadata_Temperature']) + ' batch=' + str(
            row['Image_Metadata_Batch']) + ' plate=' + str(row['Image_Metadata_Plate']) + ' well=' +
row[
                'Image_Metadata_Well'] + ' could not be processed')
        return 0, 0, 0, 0, 0, 0, 0, 0, 0, 0, 0
    m1_init = yFreq[:average].argmax()
    s1_init = m1_init / 4
    m2_init = average + yFreq[average:].argmax()
    s2_init = m2_init / 4
    m3_init = 40
    s3_init = 8
    initialGuess = numpy.array([.5, .4, m1_init, s1_init, m2_init, s2_init, m3_init, s3_init])

    bnds = ((0, 1), (0, 1), (1, 64), (0.1, 32), (1, 64), (0.1, 32), (1, 64), (0.1, 64))
    cons = ({'type': 'ineq', 'fun': lambda p: (p[2] < p[4]) - p[0] - p[1]})

    def likelihood(p):
        # prob<-function(p) p[1]*fthn(p[3],p[4])+p[2]*fthn(p[5],p[6])+(1-p[1]-p[2])*fthn(p[7],p[8])
        prob = p[0] * fthn(p[2], p[3]) + p[1] * fthn(p[4], p[5]) + abs(1 - p[0] - p[1]) * fthn(p[6],
                                                                                  p[7]) + numpy.finfo(
            numpy.float64).eps

        # L<- function(p) -sum(fr*log(prob(p)))
```

```
        return -(yFreq * [math.log(value) for value in prob]).sum()

    optimized_p = minimize(likelihood, initialGuess, method='SLSQP', bounds=bnds, constraints=cons)

    xValues = numpy.arange(0.5, 64)
    yValues_P1 = numpy.array(
        [optimized_p.x[0] * norm.pdf(x, loc=optimized_p.x[2], scale=optimized_p.x[3]) for x in xValues])
    yValues_P2 = numpy.array(
        [optimized_p.x[1] * norm.pdf(x, loc=optimized_p.x[4], scale=optimized_p.x[5]) for x in xValues])
    yValues_P3 = numpy.array(
        [(1 - optimized_p.x[0] - optimized_p.x[1]) * norm.pdf(x, loc=optimized_p.x[6],
scale=optimized_p.x[7]) for x in
         xValues])
    estimated = yValues_P1 + yValues_P2 + yValues_P3

    # chi2=((yValues-n*estimated)**2/(n*estimated)).sum()
    chi2 = chisquare(yValues, n * estimated).statistic
    sqf = (chi2 - 64 - 9) / n

    #print(optimized_p)
    #print('sqf=' + str(sqf))
    print('temp=' + str(row['Image_Metadata_Temperature']) + ' batch=' + str(
        row['Image_Metadata_Batch']) + ' plate=' + str(row['Image_Metadata_Plate']) + ' well=' + row[
            'Image_Metadata_Well'] + ' is processed')

    return optimized_p.x[0], optimized_p.x[1], optimized_p.x[0] / optimized_p.x[1], 1 -
optimized_p.x[0] - \
           optimized_p.x[1], optimized_p.x[2], \
           optimized_p.x[3], optimized_p.x[4], optimized_p.x[5], optimized_p.x[6], optimized_p.x[7], sqf


wells_data = pandas.read_csv(csv_file_to_process + ".csv", sep=',')
wells_data[
```

```
    ['DNA_Histo_P1', 'DNA_Histo_P2', 'DNA_HistoP1/P2', 'DNA_Histo_P3', 'DNA_Histo_m1',
'DNA_Histo_s1', 'DNA_Histo_m2',
     'DNA_Histo_s2',
     'DNA_Histo_m3', 'DNA_Histo_s3', 'DNA_Histo_SQF']] = wells_data.apply(process_one_well,
axis=1,
                                                           result_type="expand")

wells_data.to_csv(csv_file_to_process + extension_for_processed_file + ".csv", sep=',')
```

## References


Birmingham, Amanda; Selfors, Laura M.; Forster, Thorsten; Wrobel, David; Kennedy, Caleb J.; Shanks, Emma et al. (2009): Statistical methods for analysis of high-throughput RNA interference screens. In *Nat Methods* 6 (8), pp. 569–575. DOI: 10.1038/nmeth.1351.

Brideau, Christine; Gunter, Bert; Pikounis, Bill; Liaw, Andy (2003): Improved statistical methods for hit selection in high-throughput screening. In *Journal of biomolecular screening* 8 (6), pp. 634–647. DOI: 10.1177/1087057103258285.

Horn, Thomas; Sandmann, Thomas; Boutros, Michael (2010): Design and evaluation of genome-wide libraries for RNA interference screens. In *Genome biology* 11 (6), R61. DOI: 10.1186/gb-2010-11-6-r61.

McQuin, Claire; Goodman, Allen; Chernyshev, Vasiliy; Kamentsky, Lee; Cimini, Beth A.; Karhohs, Kyle W. et al. (2018): CellProfiler 3.0: Next-generation image processing for biology. In *PLoS biology* 16 (7), e2005970. DOI: 10.1371/journal.pbio.2005970.

Preibisch, Stephan; Saalfeld, Stephan; Tomancak, Pavel (2009): Globally optimal stitching of tiled 3D microscopic image acquisitions. In *Bioinformatics (Oxford, England)* 25 (11), pp. 1463–1465. DOI: 10.1093/bioinformatics/btp184.

Smith, Kevin; Li, Yunpeng; Piccinini, Filippo; Csucs, Gabor; Balazs, Csaba; Bevilacqua, Alessandro; Horvath, Peter (2015): CIDRE: an illumination-correction method for optical microscopy. In *Nat Methods* 12 (5), pp. 404–406. DOI: 10.1038/nmeth.3323.
